# Inositol polyphosphate-4-phosphatase type II plays critical roles in the modulation of cadherin-mediated adhesion dynamics of pancreatic ductal adenocarcinomas

**DOI:** 10.1080/19336918.2018.1491496

**Published:** 2018-08-19

**Authors:** Bin Zhang, Weidong Wang, Chonghui Li, Rong Liu

**Affiliations:** a 2nd Department of Hepatobiliary Surgery, Chinese People’s Liberation Army (PLA) General Hospital, Beijing, China; b Institute of Burns, The 1st Affiliated Hospital of Chinese PLA General Hospital, Beijing, China; c Institute of Hepatobiliary Surgery, Chinese PLA General Hospital, Beijing, China

**Keywords:** AKT, cadherins, endosomes, neoplasm invasiveness, pancreatic ductal carcinoma, INPP4B

## Abstract

The inositol polyphosphate-4-phosphatase type II (INPP4B) has been mostly proposed to act as a tumor suppressor whose expression is frequently dysregulated in numerous human cancers. To date, little is unveiled about whether and how INPP4B will exert its tumor suppressive function on the turnover of cadherin-based cell-cell adhesion system in pancreatic ductal adenocarcinomas (PDACs) *in vitro*. Here we provide the evidence that INPP4B manipulates cadherin switch in certain PDAC cell lines through a phosphorylated AKT-inactivation manner. The knockdown of INPP4B in AsPC-1 results in a more invasive phenotype, and overexpression of it in PANC-1 leads to partial reversion of mesenchymal status and impediment of *in vitro* invasion but not migration. More importantly, E-cadherin (Ecad) is enriched in the early and sorting endosomes containing INPP4B by which its recycling rather than degradation is enabled. Immunohistochemical analysis of 39 operatively resected PDAC specimens reveals it is poorly differentiated, non-cohesive ones in which the INPP4B and Ecad are partially or completely compromised in expression. We therefore identify INPP4B as an tumor suppressor in PDAC which attenuates AKT activation and participates in preservation of Ecad in endocytic pool and cellular membrane.

## Introduction

Epithelial-mesenchymal transition (EMT) is a developmental progress that endows epithelial cell with partial loss of epithelial features and gain of mesenchymal phenotype due to being transcriptionally reprogrammed. The classical event frequently seen in cancer-associated EMT is characterized by the cadherin switch between E- and N-cadherin (Ecad, Ncad), under certain circumstances, together with the varying levels of vimentin (Vim) [1]. This mesenchymal state facilitates destabilization of cell-cell adhesion complex and change of cell polarity, resulting in increased invasiveness of tumor cells [2].

The advances achieved recently shed new light on the pivotal role of phosphatidylinositol-3,4-bisphosphate (PI(3,4)P_2_) metabolism and signaling in the regulation of cancerous cellular events: motility and invasiveness [3,4]. Mounting evidences have established that PI(3,4)P_2_ dominates a distinct by-pass of the PI3K/AKT pathway. Signaling transduced by PI(3,4)P_2_ can be modulated by inositol polyphosphate 4-phosphastases type II (INPP4B), which preferentially hydrolyzes PI(3,4)P_2_ to phosphatidylinositol-3-phosphate (PI(3)P) [5]. This dephosphorylation compromises the activation of AKT, which unveils the tumor-suppressing function of INPP4B in the context of some malignancies [6,7]. The model proposed by a relevant research indicates that INPP4B promotes the serum and glucocorticoid-regulated kinase 3 (SGK3)-mediated degradation of metastasis suppressor N-myc downstream regulated 1 (NDRG1) in breast cancer harboring oncogenic mutation in PIK3CA [8]. Additionally, NDRG1 could recruit to sorting/recycling endosomes and participate in recycling of Ecad [9]. We accordingly hypothesize that INPP4B could play a role in modulating the turnover of cadherins, thereby inducing stabilization of cell-cell contact and suppression of cell invasion.

To our knowledge, the contribution of INPP4B in pancreatic ductal adenocarcinoma (PDAC) progression has not been investigated so far, while accumulating data manifest the existence of EMT-related switch between Ecad and Ncad in pancreatic cancer development [10]. Herein, we have presented the demonstration that INPP4B controls EMT marker turnover via rectifying AKT activation in the metastatic cascade of certain PDAC cell lines, and examined the association of INPP4B and Ecad expression with various clinicopathological parameters in human pancreatic tumor samples. We also demonstrate INPP4B is highly involved in endocytic and recycling dynamics of Ecad.

## Results

### INPP4B expression correlates with EMT markers of PANC-1 and aspc-1 in vitro

**Figure 1.**
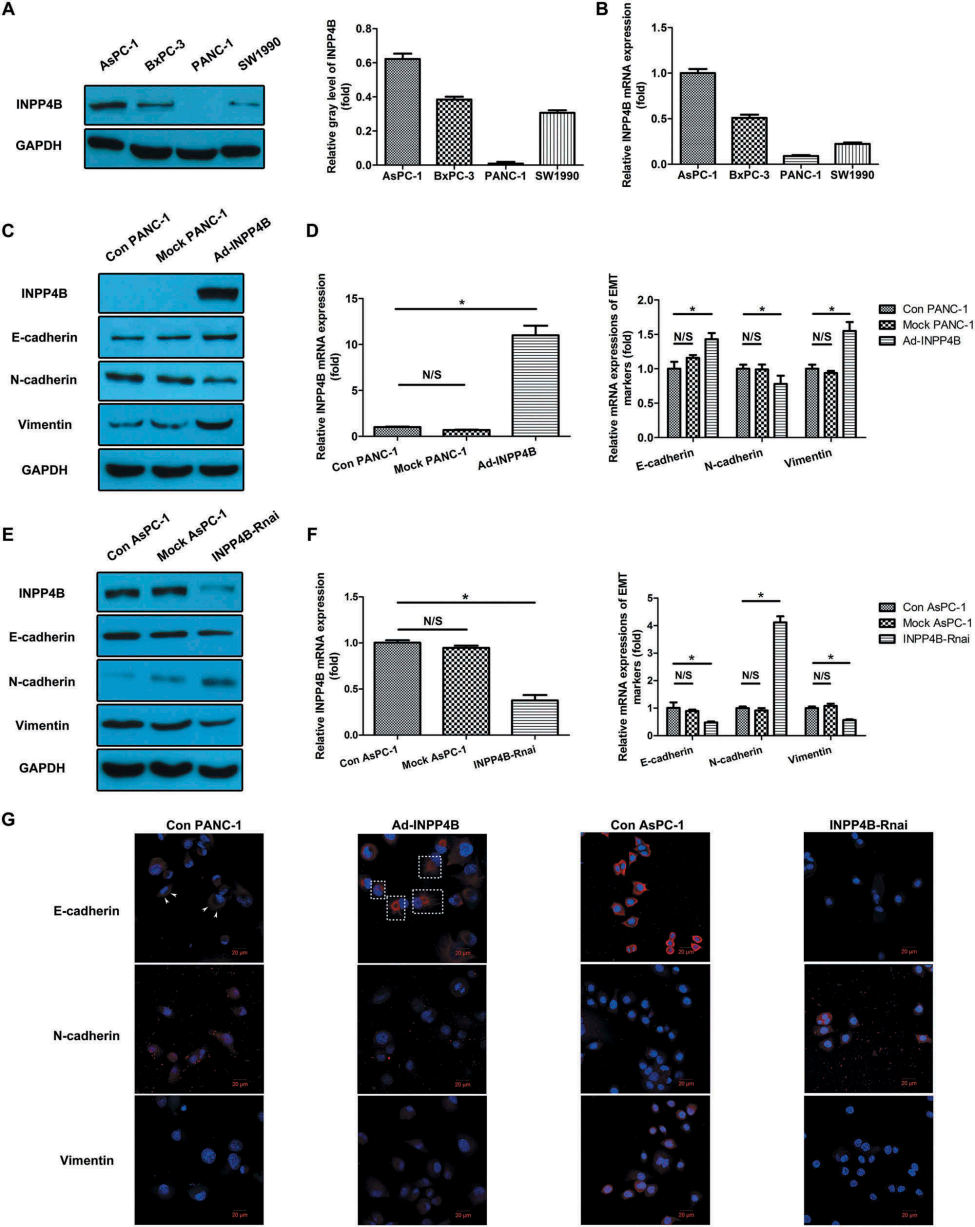
**INPP4B expression in four PDAC cell lines and its impact on EMT marker in PANC-1 and aspc-1.** Relative INPP4B expression level in four pancreatic cancer cell lines was quantified by western blot (a) and RT-qPCR (b) and listed as follows : AsPC-1> BxPC-3> SW1990> PANC-1 (All P < 0.05). The PANC-1 with relatively minimal INPP4B level was chosen for overexpression assay, and AsPC-1 with the highest INPP4B level among was selected for knockdown use. INPP4B overexpression induced upregulation of Ecad and Vim with downregulated Ncad in PANC-1 compared to that of the control in protein (c) and transcript levels (d). Knockdown of INPP4B in AsPC-1 led to reduction of Ecad and Vim with increased Ncad compared to control group (e, f). These results were further substantiated by immunofluorescence analysis for Ecad (g, top panel), Ncad (middle panel) and Vim (bottom panel) expression. Scale bar in images = 20 mm. The quantitative results of western blot and PCR were analyzed basing on gray-level ratio of target band and 2-△△_Ct_ method. GAPDH was used as housekeeping gene for all data normalization. Results show mean ± SD (n = 3). N/S and * indicate *P*> 0.05 and *P* < 0.05. The boxes and arrowheads in merge images indicate regions of interest.

We observed very low expression of INPP4B at both protein (Figure 1(a)) and mRNA levels (Figure 1(b)) in PANC-1 and SW1990 compared to AsPC-1 and BxPC-3. We selected AsPC-1 for knockdown use due to its highest INPP4B level among, while the PANC-1 was chosen for overexpression assay owing to its minimal INPP4B level. INPP4B upregulation in PANC-1 significantly downregulated the mesenchymal marker Ncad (1.28-fold reduction of transcript and 1.85-fold reduction of protein; *P *< 0.05), and upregulated the Vim (1.55-fold increase of transcript and 2.09-fold increase of protein; *P *< 0.05) and epithelial maker Ecad (1.43-fold increase of transcript and 1.59-fold increase of protein; *P *< 0.05) compared with that of the control (Figure 1(c,d), Figure S1A). Knockdown of target gene in AsPC-1 significantly decreased the Vim (1.75-fold reduction of transcript and 1.52-fold reduction of protein; *P *< 0.05) and Ecad (2.08-fold reduction of transcript and 1.61-fold reduction of protein; *P *< 0.05), with elevated Ncad level (4.12-fold increase of transcript and 3.31-fold increase of protein; *P *< 0.05) relative to the control (Figure 1(e,f), Figure S1B). These findings were further supported by the varying intensities of target protein staining when INPP4B expression was modified in immunofluorescence analysis (Figure 1(g)). The Ecad staining was originally located in cell membrane and peripheral region (Figure 1(g), arrowheads). When it was upregulated by INPP4B, both the membranous and cytoplasmic expressions were simultaneously altered (Figure 1(g), boxed areas). Although cytoplasmic expression of Ncad was also detected when cadherin switch was evident in transduced cells, few membranous expression was obviously observed, and perhaps the lack of mature cell-cell junctions could account for this phenomenon.

### INPP4B regulates cadherin switch through inhibiting phosphorylation of AKT

**Figure 2.**
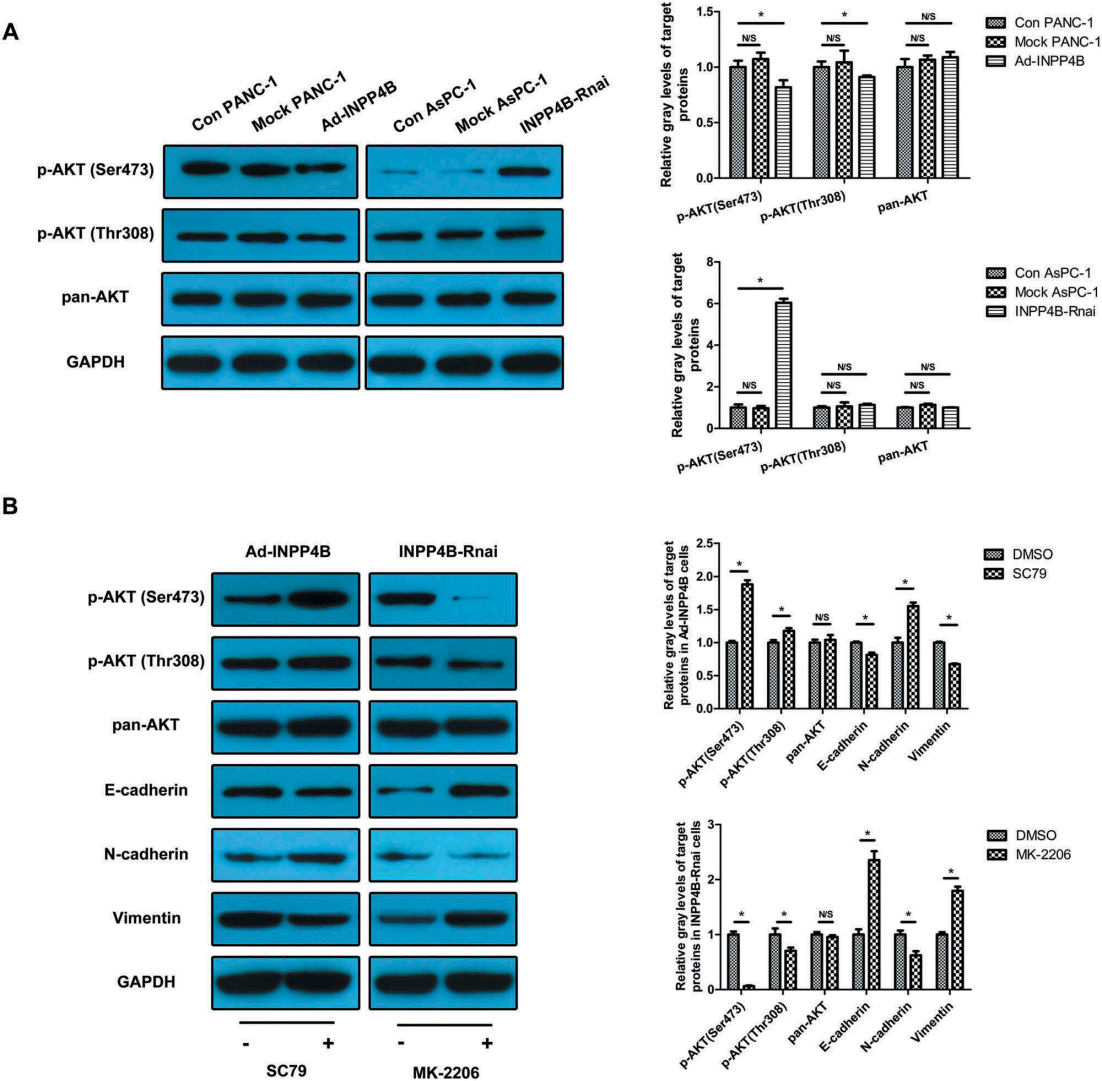
**The involvement of AKT in INPP4B-mediated EMT marker turnover** (a) Western blot analysis of transduced cell lysates showed INPP4B mainly inhibited AKT activation at Ser473 residue compared to control ones. (b) SC79 treatment on Ad-INPP4B infected PANC-1 led to a significant increase in p-AKT at Ser473 and Thr308 compared to DMSO-treated cells, which contributed to the impairment of INPP4B function on EMT marker turnover. MK-2206 treatment in INPP4B-siRNA transfected AsPC-1 simulated the effect of INPP4B on p-AKT (Ser473) and compensated for the adverse impact of INPP4B loss on cadherin and Vim expression compared to DMSO-treated cells. The results of western blot were analyzed basing on gray-level ratio of target band. GAPDH was used as housekeeping gene for data normalization. Results show mean ± SD (n = 3). N/S and * indicate *P*> 0.05 and *P* < 0.05.

In order to confirm our hypothesis that INPP4B acted as a tumor suppressor in PDAC cells through rectifying PI3K/AKT signaling, phosphorylation status of AKT was tested in Ad-INPP4B and INPP4B-Rnai group by western blot. We subsequently realized that INPP4B predominantly mediated dephosphorylation of phospho-AKT (p-AKT) at Ser473 residues. In particular, the INPP4B overexpression in PANC-1 contributed more to Ser473 dephosphorylation than to Thr308 (1.22-fold decrease vs. 1.09-fold decrease), and INPP4B knockdown in AsPC-1 only resulted in Ser473 phosphorylation and activation of AKT (6.04-fold increase; All *P *< 0.05; Figure 2(a)). To determine whether INPP4B controlled EMT-related protein turnover via inhibiting AKT activation, Ad-INPP4B infected PANC-1 was incubated in SC79 containing medium, and the INPP4B-siRNA transfected AsPC-1 was subjected to MK-2206 treatment. Immunoblot analysis showed that the INPP4B overexpression-induced reduction of Ncad, as well as increase of Ecad, was attenuated by SC79 which reactivated AKT mostly at Ser473 residue in PANC-1(1.87-fold increase vs. 1.18-fold increase). In addition, the regulatory effect of INPP4B knockdown on cadherins in AsPC-1 was also impaired by MK-2206 which mainly restrained the Ser473 phosphorylation (15.92-fold reduction vs. 1.42-fold reduction; All *P *< 0.05; Figure 2(b)). These data confirmed that INPP4B regulated cadherin expression in an AKT-dependent manner. However, INPP4B-mediated Vim expression alteration was also significantly disrupted by both the treatments, which denoted AKT signaling participated in this process but inversely regulated it versus Ncad.

### INPP4B is involved in the process of partial mesenchymal-epithelial transition (MET) or remodeling of cadherin-mediated adhesions

**Figure 3.**
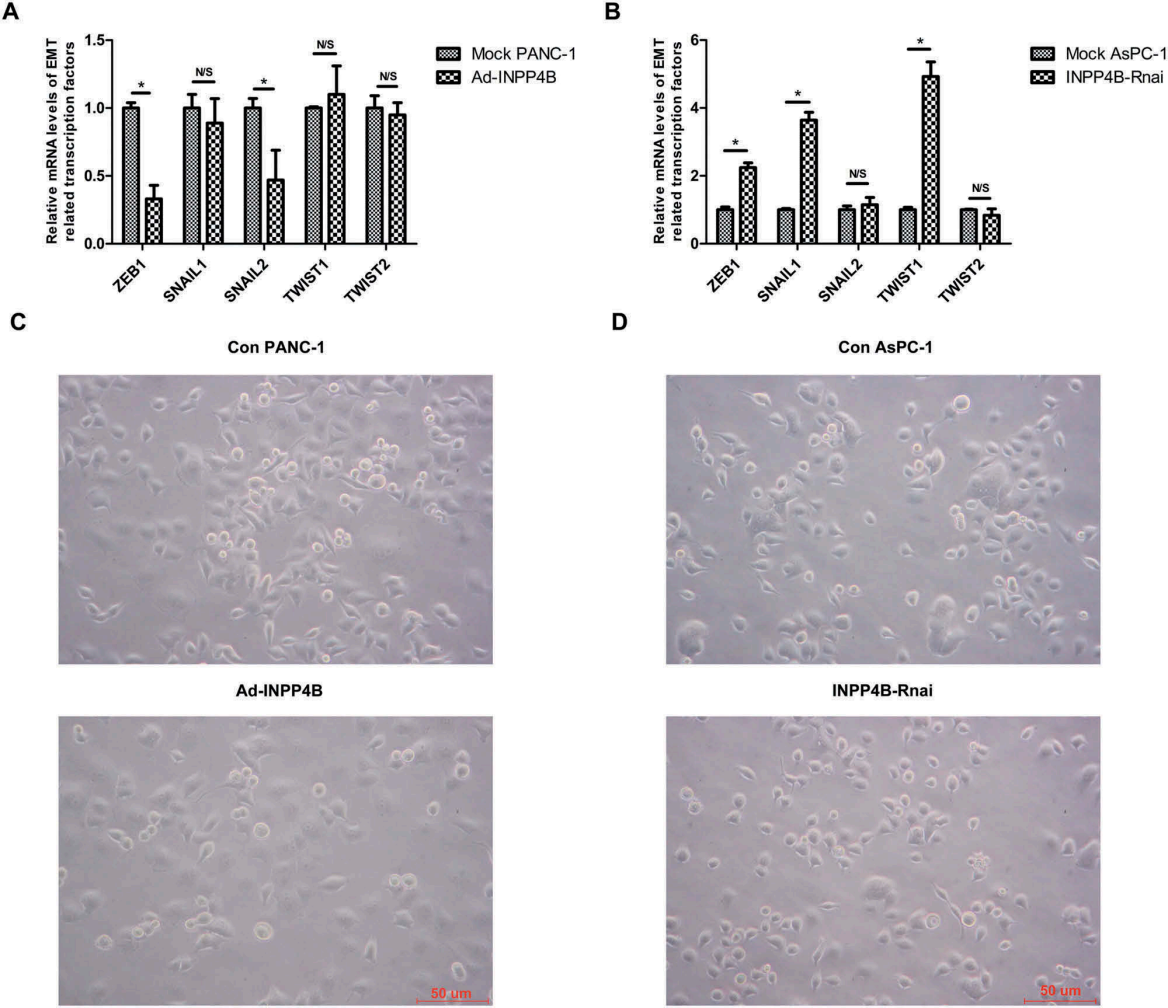
**Correlations of INPP4B expression with EMT-associated transcription factors and cell morphology** (a) Graph showing relative expression of genes (ZEB1, ZEB2, SNAIL1, SNAIL2 and TWIST) in Ad-INPP4B and INPP4B-Rnai groups compared to that in mock controls. RT-qPCR data analysis was based on 2-△△_Ct_ method and GAPDH was used as housekeeping gene for data normalization. Results show mean ± SD (n = 3). N/S and * indicate *P*> 0.05 and *P* < 0.05. (b) Phase contrast images of mock control cells and transduced cells for morphology analysis. Scale bar in images = 50 mm.

We evaluated the transcript changes of ZEB1, SNAIL1, SNAIL2, TWIST1 and TWIST2, which were considered as putative EMT-associated transcription factors, in cells with altered expression of INPP4B. RT-qPCR analysis indicated that the expression of ZEB1 and SNAIL2 were notably downregulated in Ad-INPP4B group (All *P *< 0.05; Figure 3(a)), whereas INPP4B interference in AsPC-1 resulted in a significant increase of ZEB1, SNAIL1 and TWIST1 (All *P *< 0.05; Figure 3(b)). We failed to amplify the ZEB2 fragment from these cell lines using two pairs of primers, while it was positive in TPC-1 and A498 cell lines, which suggested the scarce expression of ZEB2 in PANC-1 and AsPC-1 (data not shown). We therefore concluded that certain EMT-associated transcription factors were differentially controlled by INPP4B in PANC-1 and AsPC-1. However, the morphological characteristics of the transduced cell lines revealed by phase contrast images were not markedly different to the mock controls. Specially, mock control PANC-1 cells displayed a mesenchymal-like morphology, and Ad-INPP4B infected PANC-1 cells were still elongated in shape (Figure 3(c)). Mock control AsPC-1 cells were observed to form tight colonies. After INPP4B knockdown, cells were more scattered but without evident shape change (Figure 3(d)). Accordingly, INPP4B could only modulate cell-cell contact or aid in partial MET, and the complete reversion of mesenchymal phenotype and gain of epithelial features was hardly observed.

### INPP4B contributes to the manipulation of pancreatic cancer in vitro invasiveness

**Figure 4.**
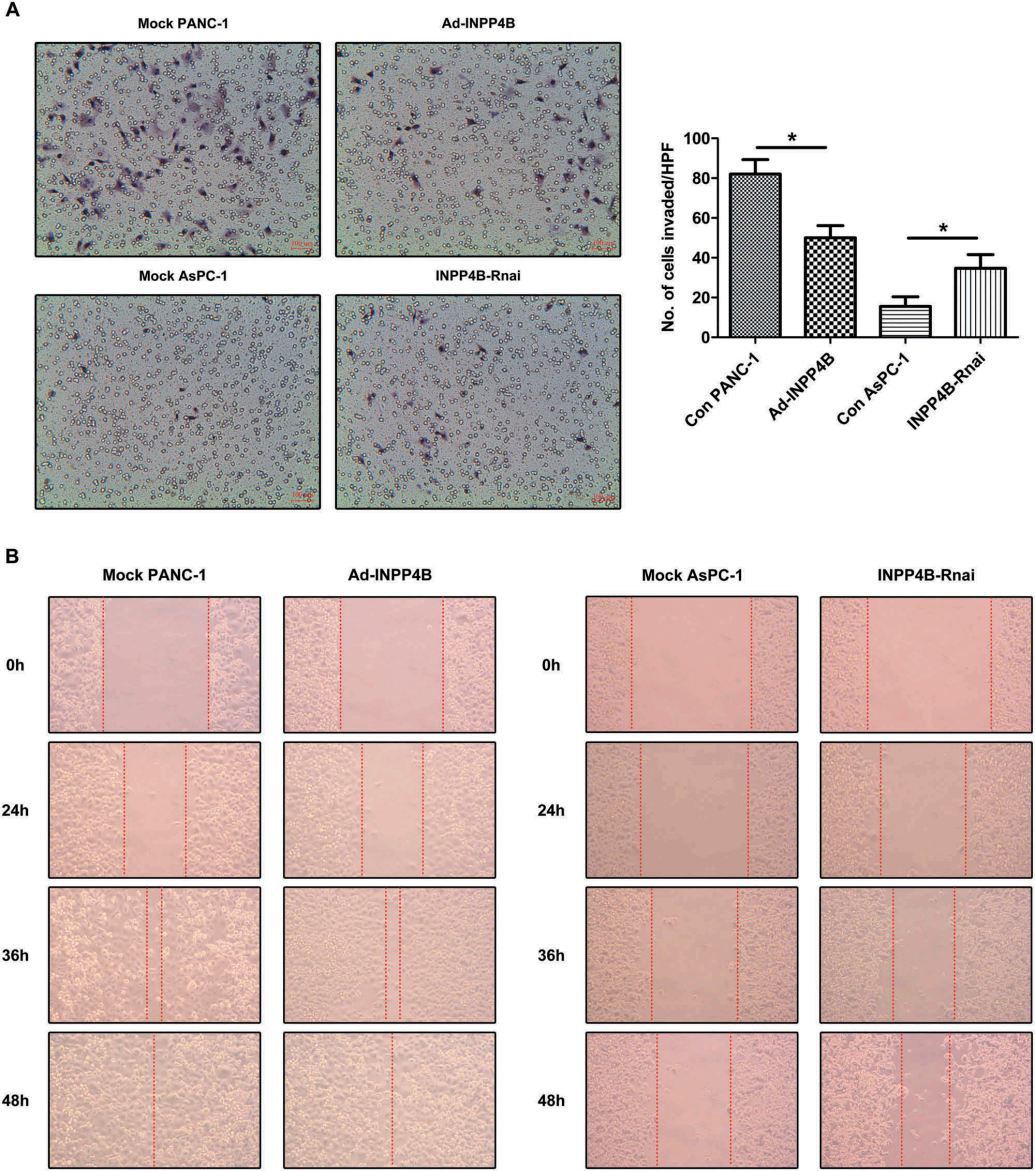
**Transwell invasion assay and wound healing assay.** (a) PANC-1 and AsPC-1 cells, untreated or undergone INPP4B upregulation/knockdown, were assessed for in vitro invasiveness through Matrigel, and the number of cells invaded through the Transwell system was recorded. (b) Representative images of wound healing assay were produced after scraping for 0 h, 24 h, 36 h and 48 h. Scale bar in images = 100 mm. Results show mean ± SD (n = 3). N/S and * indicate *P*> 0.05 and *P* < 0.05.

After 48 h of invasion, there were about 50 ± 6 cells per high power field (HPF) in Ad-INPP4B group and 34 ± 7 cells in INPP4B-Rnai group invaded through the Matrigel and Transwell membrane, comparing to 82 ± 7 in Con PANC-1 group and 16 ± 5 in Con AsPC-1 group (*P *< 0.05; Figure 4(a)). In terms of wound closing, the cell migration was allowed for 48 h and recorded in Figure 4(b). PANC-1 cells overexpressing INPP4B migrated at the similar rate as the control cells did (*P *> 0.05; Figure 4(b) left panel, Figure S1C), and the interference of INPP4B in AsPC-1 led to an acceleration in wound healing progress (*P *< 0.05; Figure 4(b) right panel, Figure S1C). These results implied that INPP4B manipulated the invasive behavior of pancreatic carcinoma *in vitro*.

### Ecad is stabilized by INPP4B in cycloheximide (chx)-treated PANC-1

**Figure 5.**
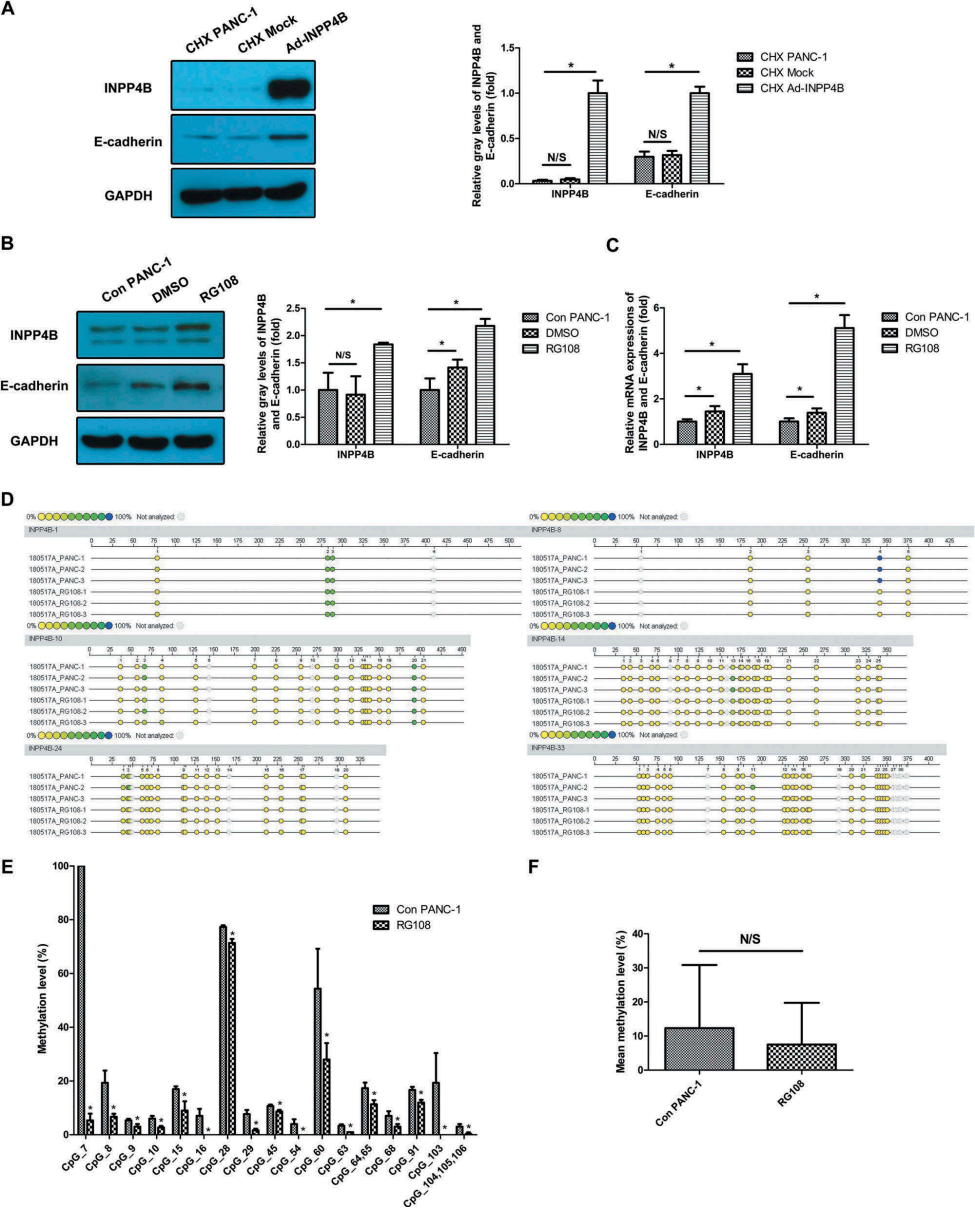
Chemicals treatment of PANC-1(a) PANC-1 cells overexpressing INPP4B were incubated in RPMI containing CHX and lysed for western blotting detection of Ecad and INPP4B. Mock infected and uninfected cells were parallelly treated with CHX and served as controls. (b, c) PANC-1 cells infected by Ad-INPP4B were treated with RG108 and probed for Ecad and INPP4B by western blot and RT-qPCR. DMSO-treated and untreated cells served as controls. The quantitative results of western blot and PCR were analyzed basing on gray-level ratio of target band and 2-△△_Ct_ method. GAPDH was used as housekeeping gene for all data normalization. (d) Sequenom MassARRAY platform was used for the quantitative methylation analysis. The colors of each circle represent the methylation level of each corresponding CpG unit. Quantitative methylation analysis results are shown in a color scale: yellow (0% methylation), green (50% methylation), and dark blue (100% methylation). The white circles represent the missing data at a given CpG site. (e) The significantly changed methylation levels of certain CpG units were shown. (f) Mean methylation percentage of total CpG sites evaluated in the control group and RG108 group was compared. Results show mean ± SD (n = 3). N/S and * indicate *P* > 0.05 and *P* < 0.05.

PANC-1 overexpressing INPP4B showed a less decrease in Ecad protein levels as compared to mock infected and uninfected cells (*P* < 0.05; Figure 5(a)) when treated with a protein translation inhibitor, CHX. This illustrated Ecad could be stabilized by INPP4B.

### RG108 upregulates INPP4B and ecad in PANC-1

Treatment of PANC-1 with RG108 heightened INPP4B expression approximately 3.09-fold in transcript level and 1.84-fold in protein level relative to control. There was also a 5.11-fold increase in Ecad transcript level and 2.18-fold increase in protein level (All *P *< 0.05; Figure 5(b,c)), more than we anticipated, which could be attributed to the upregulation of INPP4B and the revision of intrinsic methylation of CDH1 gene promoter in PANC-1 [11]. An increase of both the INPP4B and Ecad was also detected in DMSO-treated cells, although the statistical significance of this observation was borderline (0.01 < *P *< 0.05), which we could reasonably ascribe to the epigenetic impact of DMSO on cells *in vitro* [12].

### Upregulated INPP4B expression after RG108 treatment is not initiated by its promoter region demethylation

RG108 can suppress DNA methyltransferase (DNMT) as well as increase INPP4B expression in PANC-1. Thus, we investigated whether the INPP4B downregulation in PANC-1 was due to abnormal promoter demethylation or not. Four CpG enriched or CpG island containing regions spanning the promoter region and exon 1 in the INPP4B gene were amplified and evaluated for methylation level respectively (Figure S2). The methylation scale of almost 85% of the total CpG units evaluated was below 20%. The highest methylation level (100%) was only found at the 7th CpG sites (Figure 5(d)). When treated with RG108, though the methylation levels of certain CpG units declined (All *P *< 0.05; Figure 5(e)), the average methylation percentage across total CpG sites evaluated was not significantly reduced (*P *> 0.05; Figure 5(f)). Therefore, RG108 upregulated INPP4B expression without apparently inducing DNA demethylation of its promoter region. It might be ascribed to the intrinsic hypomethylation of overall INPP4B promoter region in PANC-1.

### INPP4B actively participates in the recycling of ecad

**Figure 6.**
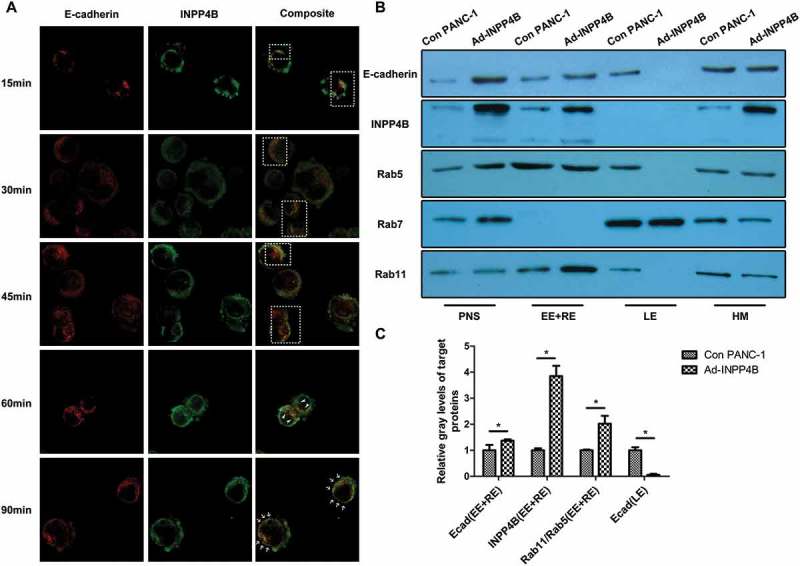
**INPP4B co-localizes and co-fractionates with endocytic ecad** (a) The dynamics of Ecad recycling relating to INPP4B in RG108-treated PANC-1 cells were investigated via immunofluorescence analysis at different time points after calcium chelation. Immunostaining of Ecad (red) and INPP4B (green) were shown in separate channels. The boxes, arrows and arrowheads in merge images indicate regions of interest. (b) PANC-1 cells were subjected to a discontinuous sucrose density gradient centrifugation, and the proteins from resulting cell fractions were loaded for western blot. (c) The Ecad and INPP4B blots were normalized to Rab5 and 11 in EE and RE, as well as Rab7 in LE, to reveal the endosomal contents respectively. The Rab11/Rab5 ratio in EE and RE was calculated to reflect the relative recycling activity relating to INPP4B overexpression. PNS: postnuclear supernatant; EE: early endosomes; RE: recycling endosomes; LE: late endosomes; HM: heavy membranes. The results of western blot were analyzed basing on gray-level ratio of target band. Results show mean ± SD (n = 3). * indicate *P* < 0.05.

In order to avoid false-positive result from INPP4B overexpression in double-labeling immunofluorescence of Ecad and INPP4B, the PANC-1 cells were pretreated with RG108 rather than Ad-INPP4B to discern the possible co-localization. Confocal laser scanning analysis revealed that punctate staining for INPP4B was observed at the cytomembrane and peri-membranal region (Figure 6(a), middle column). INPP4B was detected co-localized with recycling Ecad as time elapsed after calcium chelation (co-localizing green and red pixels in composite plan were pseudocolored in yellow). In detail, as the cells spread, structures positive for both the proteins resolved into tightly clustered morphology (Figure 6(a); boxed areas) and gradually diffused freely along the membrane if no mature cell-cell adhesive structures took shape (Figure 6(a); arrows). Clusters of the two proteins laterally moved to the cell junctions in a constrained fashion if two cells reached confluence (Figure 6(a); arrowheads).

### INPP4B increases ecad at early and recycling endosomes and suppresses its late endosomal lodging

Western blot of equal amount of proteins from resulting cellular fractions showed that the two target proteins were mainly precipitated from Rab5 and 11 positive parts (Figure 6(b)). Rab5 was loaded as marker for early endosomes (EE), Rab11 for recycling endosomes (RE) and Rab7 for late endosomes (LE) [13,14]. When normalized by Rab5 and 11 in EE and RE fractions, the early and sorting pools of Ecad were enriched by INPP4B overexpression comparing to that of the control. We also normalized the corresponding Ecad to Rab7 in LE fraction and observed that the degradation pools of Ecad in Ad-INPP4B cells decreased. Moreover, the Rab11/Rab5 ratio in EE and RE fractions was elevated in Ad-INPP4B cells which could be interpreted as INPP4B overexpression promoted transport and recycling of membrane protein (All *P *< 0.05; Figure 6(c)). HM fractions were strongly positive for both the proteins in Ad-INPP4B group, which might in part ascribe to the upregulated expression of them on cytomembrane when taking into account the immunofluorescence result.

### INPP4B and ecad expression in clinically resected pancreatic tumor specimens

**Figure 7.**
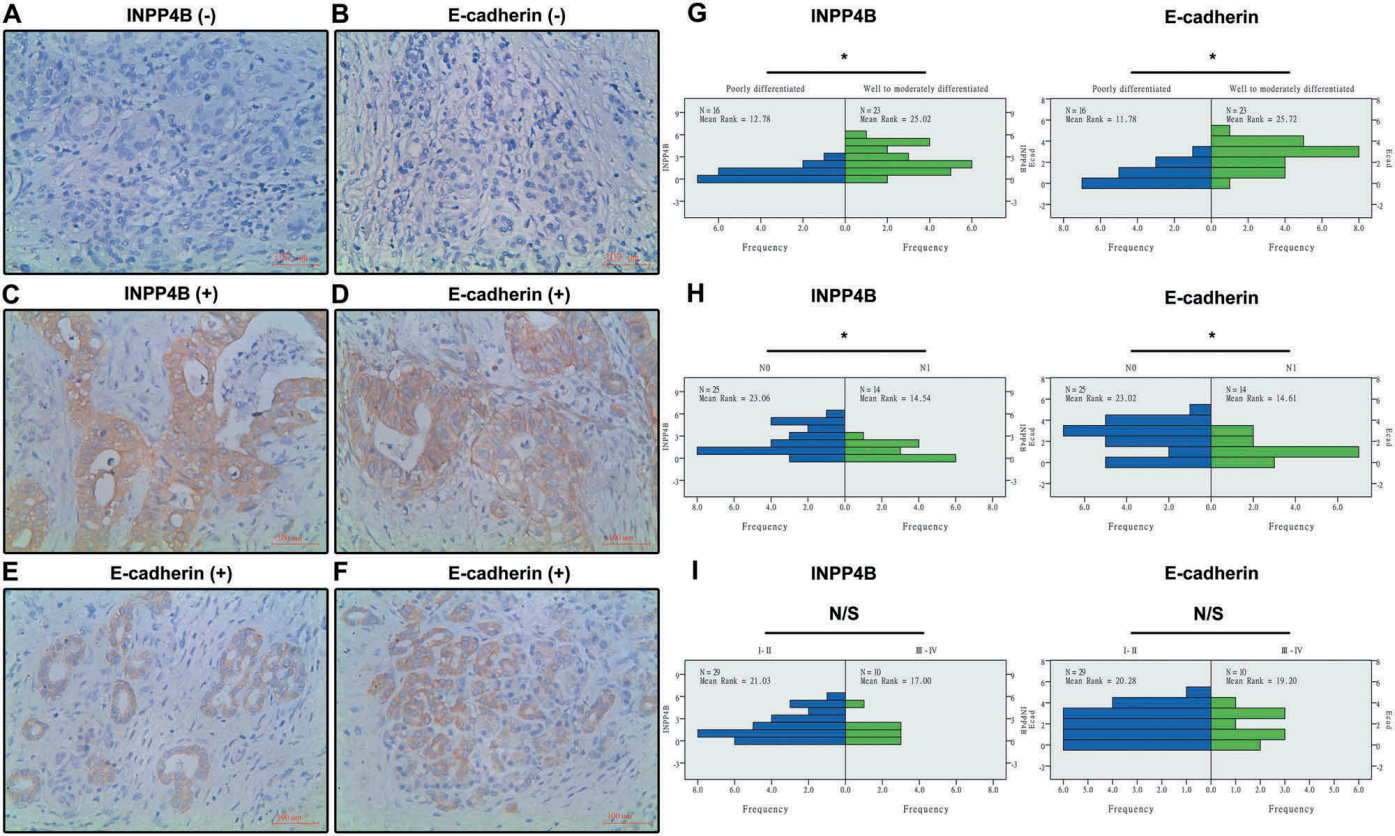
**Expression of INPP4B and ecad in pancreatic tumor specimens detected by immunohistochemistry**. Shown were representative photomicrographs (×400) of negatively (a, b) and positively stained specimens (c-f). Epithelial cancer cells with more cytoplasmic expression of Ecad than membranous expression (e), and cells with only membranous loss of Ecad (f) were detected. Relevance of target protein expression to tumor grade (g), lymph node metastasis status (h) and TNM stage (i) of tissues was expounded by Mann-Whitney U test. N0 indicates no lymph node metastasis. N1 indicates regional lymph node metastasis. Scale bar in images = 100 mm. Results show mean ± SD (n = 3). N/S and * indicate *P* > 0.05 and P < 0.05.

Positively stained INPP4B and Ecad were mainly located in the cytoplasm of cancerous ductal cells (Figure 7(c-f)). We did observe some cancer cells with more cytoplasmic expression than membranous expression (Figure 7(e)), and cells with only membranous loss of expression (Figure 7(f)), which would indicate malposition of Ecad. The median age at diagnosis of the 39 patients was 57.3 ± 7.2 years. Correlations of the INPP4B and Ecad staining index with pathological parameters were listed in Figure 7(g-i). The staining of INPP4B expression inversely correlated with tumor grade and lymph node metastasis status, and a similar trend was also noted for that of Ecad (All *P *< 0.05; Figure 7(g,h)). A Spearman rank correlation analysis indicated a positive correlation between the two proteins in PDAC (r = 0.52, *P* < 0.05). However, TNM stage is a macroscopic and anatomy-dependent system, for instance the involvement of celiac axis, which would not truly or fully reflect the cancerous behavior of certain sets of pancreatic tumors, and thus we found no significant relevance of target protein expression to TNM stage (*P *> 0.05; Figure 7(i)).

## Discussion

INPP4B is initially identified as a tumor suppressor due to its negatively regulatory effects on PI3K/AKT pathway which outlines a network that steers important biological processes such as cell adhesion and migration, all of which are disrupted in several types of cancers [15]. Cadherins are a class of type-1 transmembrane glycoproteins that function as calcium-dependent junction to bind cells together. Loss of Ecad in intercellular contacts is believed to be the first key step in development of cancer-related EMT [16]. In this report, overexpression of INPP4B in PANC-1 resulted in a raised level of epithelial maker Ecad and a decrease in mesenchymal marker Ncad through dephosphorylating p-AKT mainly at Ser473 residues, which antagonized *in vitro* invasion without affecting their overall migratory capacity. On the other hand, the silencing of target gene in AsPC-1 cells promoted cadherin switch from Ecad to Ncad depending on p-AKT upregulation and led to increased invasion and migration behavior. These data indicated INPP4B was an upstream regulator of both Ecad and Ncad in these pancreatic cancer cells. AKT activation depends on the availability of PI(3,4)P_2_ and phosphatidylinositol-3,4,5-trisphosphate (PI(3,4,5)P_3_). The INPP4B substrate PI(3,4)P_2_ contributes predominantly to Ser473 phosphorylation, whereas PI(3,4,5)P_3_, substrate of phosphatase and tensin homolog (PTEN), contributes mostly to Thr308 phosphorylation [17]. We speculated that INPP4B hydrolyzed PI(3,4)P_2_ to antagonize the hyperactivation of oncogenic PI3K/AKT signaling, which could provide an explanation for our findings in PDAC. However, the reciprocal feedback between PTEN, SHIP and INPP4B should be considered in future.

The role of EMT in the progression of epithelial carcinomas and their metastatic dissemination was debated, because in most cancers the full conversion of epithelial/mesenchymal state rarely existed [1,18]. Unlike many previous works, Vim was oppositely regulated versus Ncad by INPP4B in PANC-1 and AsPC-1 cells. The limited variation of Vim in our study could not reach that far as overexpression do, which we thought would be a physiological response to INPP4B alternation rather than pathologic effect and required further investigation. This unconventional case was also noticed in angiomyolipoma cells and interpreted by TSC2/mTOR signaling deficiency [19]. Additional approaches were utilized to verify the potential link between INPP4B and EMT as a pathophysiological process, including light microscopy, confocal fluorescence imaging and RT-qPCR analysis of EMT-associated transcription factors. The morphologic characteristic of PDAC cells observed under phase contrast microscope was not significantly changed when INPP4B expression altered, while the fluorescence intensity of EMT-related protein staining was apparently influenced respectively. The scanty cytoplasm and large nucleus of PANC-1 and AsPC-1 made it hard to discern whether the cell shape seen was really unchanged or not. It was ever reported that the nuclear factors of SNAIL, ZEB and TWIST families exerted a strong transcriptional control on EMT, among which the SNAIL, ZEB1, and ZEB2 repressed Ecad expression by binding directly to the E-box sequences of the CDH1 promoter [20]. Our data suggested only certain ones of them were robustly regulated by INPP4B in PDAC cells. Altogether, these findings confirmed a link between the expression of INPP4B and partial reversion of mesenchymal status or just the cadherin switch in PANC-1 and AsPC-1. The model we proposed was not a perfect EMT/MET, but at least a transient regulation of cell-cell adhesion structures. Perhaps inhibition of cell proliferation or proteases that degraded extracellular matrix was involved in INPP4B-oriented suppression of PANC-1 invasion but not migration.

The deterministic indicator role of Ecad in differentiation and invasiveness of various PDAC cell lines, including differentiated ones (Capan-1, Capan-2 and DAN-G), intermediate state (Hs 766T) and undifferentiated cells (MIA PaCa-2), was disclosed by a previous investigation [21]. Meanwhile, PANC-1, started from a human undifferentiated pancreatic carcinoma [22], was proved relatively minimal in INPP4B level among the four PDAC cell lines we selected. We also detected the depressed amount of INPP4B within the moderately differentiated cell line SW1990 [23]. The higher level of target protein was confirmed in BxPC-3 (originated from a moderately well differentiated foci without evidence of metastasis) and AsPC-1 (the ascitic tumor cells of a moderately well differentiated PDAC sufferer with no metastasis to internal organs of nude mice *in vivo* tumorigenicity test) [24,25]. In view of this finding, we sought to characterize the co-expression of INPP4B and Ecad in surgically resected pancreatic cancer tissues if existed, and correlate them respectively with lymph node metastasis status and tumor differentiation grade. We conclusively demonstrated that it was poorly differentiated, non-cohesive ones in which the INPP4B and Ecad were partially or completely compromised in their expression. Meanwhile, a heterogeneity in staining extent of tumor cells was found in a given individual, and the presence of a transitional phenotypic state might account for this phenomenon.

Ecad has a half life of 5 h-10 h [26]. It is internalization by endosomes and subsequent lysosomal or proteasomal degradation or recirculation back to the cell surface that takes the center stage in the metabolism of Ecad. This vesicular to and fro movement among cell surface and endosomes provides a mechanism for manipulating the availability of Ecad for cell-cell junction in tumor progression [27]. Interestingly, several studies report that PI(3)P is mainly aggregated in EE while PI(3,5)P_2_ at late endocytic compartments [28]. Aside from the given notion that INPP4B favorably mediated the turnover of PI(3,4)P_2_, we observed a greater effect on Ecad protein (1.6-fold increase) than on mRNA level (1.43-fold increase) when INPP4B was upregulated in PANC-1. So we hypothesized and aimed to provide the evidence that INPP4B could be an Ecad recycling participator recruiting to EE and RE. For this purpose, live cell labeling and recycling assay was conducted and intense stains for both the two proteins were seen at cell membrane and peripheral region. The EE was a dynamic compartment encompassing thin tubules (approximately 60 nm diameter) and large vesicles (around 300–400 nm diameter). Some separated tubular structures resolved into RE and distributed close to the centrosome for latter transport depending on microtubules [29]. Coincidently, subcellular location of INPP4B was predicted to be centrosome, cell membrane and intracellular compartment according to the Human Protein Atlas [30], which further substantiated our findings. As showed by boxed areas of Figure1(g), Ecad staining transformed into a filamentous appearance when INPP4B was overexpressed. Besides, functions of Vim contributed to the construction of cytoskeleton architecture within cells by interacting with microtubules [31]. These data allowed us to suggest that Vim should be motivated and recruited as a candidate for cross-bridging microtubules and Ecad containing vesicles if INPP4B was upregulated [32,33].

To date, little evidence of INPP4B inactivating mutations or deletions in human cancers has been provided [8]. Treatment of thyroid cancer cell lines with 5-Aza-2ʹ-deoxycytidine (5-Aza-CdR), which inhibited DNA methylation, led to INPP4B upregulation [34]. RG108 was a DNMT inhibitor and was proved to be efficient in reactivation of epigenetically silenced tumor suppressor genes without indications for cytotoxicity comparing to 5-Aza-CdR [35,36]. PDAC cell lines often exhibited higher CpG island hypermethylation frequency than clinically resected PDAC tissues [37]. It was postulated that INPP4B loss in PANC-1 would be due to aberrant gene promoter methylation. We then compared the methylation status of INPP4B promoter region in control and RG108 treated cells by MassARRAY methylation analysis but the result refuted our hypothesis. Therefore, INPP4B upregulation in RG108 treated cells was potentially indirect, mediated via the increase of yet to be defined transcription factors. RG108 treatment enabled the immunostaining of both the proteins in PANC-1 to monitor the recycling dynamic of Ecad relating to INPP4B, and might therefore propose an effective therapeutic agent for PDAC of INPP4B/Ecad loss or downregulated phenotype.

The relatively moderate reduction of Ecad in CHX-treated Ad-INPP4B group than in control denoted that INPP4B directly or indirectly stabilized the Ecad pool due to increasing its longevity within cells, perhaps via endocytosis and recycling pathway. Afterwards, a discontinuous sucrose density ultracentrifugation was employed to reveal the endosomal protein burden of both the INPP4B and Ecad in PANC-1. The result manifested INPP4B co-localized with Ecad in EE and RE which were bound to renew the integral membrane proteins, but not in LE destined for degradation. One of the causative mechanisms underlying the Ecad downregulation in PANC-1 may be that INPP4B loss increases the PI(3,4)P_2_ pool and prolongs AKT2 signaling on the endocytic membranes, followed by vesicular transport of protective cargoes to LE for lysosomal or proteasomal degradation [9,38]. However, we did not further purify RE from target fractions via immunoisolation using Rab11 antibody bound to immunomagnetic, which confined the level of evidence.

AsPC-1 was originally prepared from the floating cells in cancerous ascites [25], which would mimic the circulating tumor cells (CTCs) in bloodstream to some extent. Comparatively speaking the anti-oncogenic feature of INPP4B was the case in primary tumor, however, it might be upregulated in a subgroup of CTCs and dormant micrometastases, which promoted Ecad re-expression in aiding anchoring and proliferation. A significant proportion of CTC platforms use antibodies conjugated immunomagnetic beads or nanoparticles to enrich CTCs basing on the expression of cancer-specific antigens [39]. If INPP4B is absent on benign blood cells and expressed by epithelial tumor-derived cells on cytomembrane when travelling [40], it will help in capturing a subpopulation of pre-colonizing CTCs. To this end, core needle biopsy of metastatic foci and blood sample collection for CTCs enrichment are in demand for further investigation.

This study provides the first evidence that INPP4B oppositely regulates the expressions of Ecad and Ncad in a subset of PDAC cell lines in an AKT dependent manner. It functions as a tumor suppressor that inhibits AKT activation and suppresses *in vitro* invasiveness of PANC-1 and AsPC-1. Promoter region hypermethylation is not a mechanism for the silencing of INPP4B in PANC-1. Expression deficiency of Ecad and INPP4B are detected in some of the high-grade or non-cohesive human PDAC specimens. Moreover, INPP4B is involved in endocytic trafficking pathway, *inter alia* the EE and RE in which cargo protein Ecad ‘revives’. The view that INPP4B are implicated in cell-cell adhesion renewal may aid in the development of novel INPP4B-based signature for prognosis and eventually a more refined anticancer treatment.

## Materials and methods

### Cell culture and transduction

The human PDAC cell lines, AsPC-1, BxPC-3, PANC-1 and SW1990, were purchased from the National Infrastructure of Cell Line Resource (Beijing, China). All cells were maintained in DMEM (HyClone) or RPMI (HyClone) supplemented with 10% FBS (Gibco), 100 U/ml penicillin and streptomycin (Gibco), and incubated at 37 ℃ in a humidified atmosphere containing 5% CO_2_. For overexpression of INPP4B, cells were infected by a recombinant adenovirus Ad-INPP4B (Genechem) at the indicated multiplicity of infection. For knockdown purpose, a siRNA targeting the coding region of INPP4B (Gen-Bank Accession Number: NM_001101669) was chemically synthesized (GenePharma) and transfected into cells with Lipofectamine® 2000 Reagent (Invitrogen). Mock controls were transduced by vacant adenovirus or a non-targeting siRNA. Cells above were harvested 24 and 48 h post treatment for RT-qPCR and immunoblotting separately.

### RT-qPCR

The cDNA was synthesized using Revert Aid RT Kit (Thermo Fisher Scientific) according to manufacturer’s instructions. A total of 1 μg RNA was used for cDNA synthesis. RT-qPCR was performed on a Master cycler Ep Realplex (Eppendorf) using Fast SYBR® Green Master Mix (Invitrogen) with primers described in the supplemental material. PCR cycling conditions included initial denaturation at 95 °C, followed by 35 cycles of denaturation for 15 s at 95 °C, annealing for 30 s at 60 °C-64 °C and extension for 30 s at 72 °C. The comparative cycle threshold (2^−ΔΔCt^) method mapped the relative expression of target gene. GAPDH was used as housekeeping gene for all data normalization.

### Immunoblotting

Cells were lysed in RIPA buffer (Solarbio) supplemented with protease and phosphatase inhibitor cocktail (Sigma-Aldrich) and sonicated. The protein concentration was determined using the BCA Protein Assay Kit (Beyotime) and western blot was performed essentially. Target bands were detected by the following monoclonal antibodies: anti-INPP4B (1:500; #14,543), anti-Ecad (1:500; #3195), anti-Ncad (1:500; #13,116), anti-vimentin (1:1000; #5741), anti-GAPDH (1:1500; #5174), anti-pan-AKT (1:1000; #4691), anti-phospho-AKT (Ser473, Thr308; both 1:1000; #4060, #13,038) antibodies and HRP-conjugated secondary antibody (1:2000; #7074) were purchased from Cell Signaling Technology (CST). The gray-level of target band relative to GAPDH in each group was established by Quantity One software (Bio-Rad).

### Chemicals treatment

Cells transduced by Ad-INPP4B or siRNA were grown in the presence or absence of AKT activator SC79 (5 μM; ApexBio) or inhibitor MK-2206 (10 μM; ApexBio) for 24 h before harvested for western blot analysis. To inhibit protein translation, Ad-INPP4B and mock infected PANC-1 cells were treated with 10 μM CHX (Amresco) at 48 h post infection, and probed for INPP4B and Ecad via western blotting at 7 h post treatment. We then exposed PANC-1 to 100 μM RG108 (ApexBio) for 72 h through diluting the stock solution (RG108/DMSO 50 mM) in culture medium. For control purpose, PANC-1 was exposed to 0.2% DMSO only. Transcript and protein of INPP4B and Ecad in control versus RG108-treated PANC-1 cells were measured subsequently.

### Massarray quantitative methylation analysis

DNA samples from the control and RG108-treated PANC-1 cells were subjected to bisulfite modification using EZ DNA Methylation Gold Kit (Zymo Research). Six pairs of primers for methylation analysis were designed using Methprimer (http://www.urogene.org/methprimer/) and listed in the supplemental material. Sequenom MassARRAY platform (CapitalBio; Beijing, China) was used to analyze INPP4B methylation quantitatively (Gen-Bank Accession Number: NM_001101669). Mass spectra were obtained via MassARRAY Compact MALDI-TOF (Sequenom) and methylation ratios were generated using the Epityper software (Sequenom).

### Immunofluorescence microscopy

To further substantiate the findings of immunoblotting, all cells were re-plated on fibronectin-coated coverslips and cultured for 24 h. They were then fixed with 4% paraformaldehyde and permeabilized with 0.5% Triton X-100. Coverslips were washed with PBS and then incubated with specific first antibodies respectively, followed by Alexa Fluor 594-conjugated anti-rabbit secondary antibody (Thermo Scientific, #A11012) labeling. The cells were then reacted with DAPI for nuclear staining. For live cell labeling and recycling experiment, RG108-treated PANC-1 cells were labeled with mouse antibody against Ecad (CST, #5296) on ice and subjected immediately to calcium chelation by addition of 0.02% EDTA. They were then restored back to complete medium at 37 ℃ and 5% CO_2_ environment. Once anchored on coverslip, cells were fixed at different time points and probed for INPP4B with rabbit antibody (CST, #14,543), followed by Alexa Fluor 488 and 594-conjugated secondary antibodies (CST, #4412, #8890) labeling. All fluorescence images were acquired under an alpha Plan-Fluarobjective lens of Zeiss LSM 510 confocal microscope.

### Endosome purification

The PANC-1 cells were scraped off the plate and homogenized in the sucrose solution (8.5% sucrose, 3 mM imidazole, with protease inhibitor cocktail). After removing of nuclei and cell debris, postnucelar supernatant (PNS) was subjected to sucrose gradient centrifugation. Target cell fractions were collected as previously described [9,34]. Fractions at the 25%-35% interphase contained RE and EE, and LE were recovered from uppermost portion of 25% phase. Heavy membranes (HM), recovered from lowest interphase, were consisted of Golgi apparatus, endoplasmic reticulum and plasma membrane. Proteins from the organelle fractions above were precipitated with methanol/chloroform for western blot analysis.

### Transwell invasion assay and wound healing assay

Transwell inserts (Corning) with 8 μm pore polycarbonate membrane were coated with Matrigel (BD Biosciences). Cells suspended in medium containing 2% FBS were added to the upper chamber of Transwell system, and migration was allowed to proceed towards the lower section filled with complete medium for 48 h at 37 ℃. Invasion was determined by counting those cells that traversed the cell-permeable membrane. For wound healing assay, cells were grown in a 6-well plate containing complete medium until a monolayer was formed. After scraping with a 10 μl tip, cells were incubated in low serum medium (2% FBS) for 48 h. The 0 h images were taken right after wound creation by an inverted epi-fluorescence microscope. Wound healing percentage of transduced cells was measured by Image J software and normalized to controls.

### Tissue collection and immunohistochemistry analysis

The study included 39 PDAC suffers who were diagnosed and received surgical treatment in the 2nd Department of Hepatobiliary Surgery, Chinese PLA General Hospital, whose archival paraffin-embedded pathological materials were available for immunohistochemical analysis. They were re-staged postoperatively according to the American Joint Committee on Cancer (AJCC) 2010 staging system. Clinical parameters included lymph node metastasis status, tumor differentiation grade and TNM stage. No patients underwent chemotherapy or radiation therapy prior to surgery. 5 μm sections were immunostained essentially and examined by senior pathologist who was blind to clinical data of the patients. A staining index was determined by adding together the scores for staining intensity (0: no color; 1: light yellow; 2: light brown; 3: brown) and percentage of positively-stained pancreatic tumor cells (0: < 5%; 1: 5–25%; 2: 26–50%; 3: 51–75%; 4: > 75%) apart from the fiber composition. For co-expression and prognosis analysis, correlations between scores of INPP4B and Ecad, as well as pathological parameters, were analyzed.

### Statistical analysis

Each data point was displayed as the mean ± standard deviation of at least three independent biological experiments. Statistical tests used included the Student’s *t*-test, Mann-Whitney U test and Spearman rank correlation analysis. A *P*-value < 0.05 was considered to have statistical significance.

## References

[1] Christofori G. New signals from the invasive front [Research Support, Non-U.S. Gov’t Review]. Nature. 2006 5 25;441(7092):444–450. PubMed PMID: 16724056; eng. . 16724056 10.1038/nature04872

[2] Thompson EW, Newgreen DF, Tarin D Carcinoma invasion and metastasis: a role for epithelial-mesenchymal transition? [Research Support, Non-U.S. Gov’t Review]. Cancer Res. 2005 7 15;65(14):5991–5; discussion 5995 PubMed PMID: 16024595; eng. . 10.1158/0008-5472.CAN-05-0616 16024595

[3] Fukumoto M, Ijuin T, Takenawa T PI(3,4)P2 plays critical roles in the regulation of focal adhesion dynamics of MDA-MB-231 breast cancer cells. Cancer Sci. 2017 5;108(5):941–951. PubMed PMID: 28247964; PubMed Central PMCID: PMC5448597. eng. . 28247964 10.1111/cas.13215 PMC5448597

[4] Tsujita K, Itoh T Phosphoinositides in the regulation of actin cortex and cell migration [Research Support, Non-U.S. Gov’t Review]. Biochim Biophys Acta. 2015 6;1851(6):824–831. PubMed PMID: 25449647; eng. . 25449647 10.1016/j.bbalip.2014.10.011

[5] Gewinner C, Wang ZC, Richardson A, et al Evidence that inositol polyphosphate 4-phosphatase type II is a tumor suppressor that inhibits PI3K signaling. Cancer Cell. 2009 8 4;16(2):115–125. PubMed PMID: 19647222; PubMed Central PMCID: PMC2957372. eng. 19647222 10.1016/j.ccr.2009.06.006 PMC2957372

[6] Hodgson MC, Deryugina EI, Suarez E, et al INPP4B suppresses prostate cancer cell invasion [Research Support, N.I.H., Extramural Research Support, Non-U.S. Gov’t]. Cell Communication and Signaling: CCS. 2014 9;25(12):61 PubMed PMID: 25248616; PubMed Central PMCID: PMC4181726. eng. . 25248616 10.1186/s12964-014-0061-y PMC4181726

[7] Kofuji S, Kimura H, Nakanishi H, et al INPP4B Is a PtdIns(3,4,5)P3 Phosphatase That Can Act as a Tumor Suppressor [Research Support, Non-U.S. Gov’t]. Cancer Discov. 2015 7;5(7):730–739. PubMed PMID: 25883023; eng. 25883023 10.1158/2159-8290.CD-14-1329

[8] Gasser JA, Inuzuka H, Lau AW, et al SGK3 mediates INPP4B-dependent PI3K signaling in breast cancer [Research Support, N.I.H., Extramural Research Support, Non-U.S. Gov’t Research Support, U.S. Gov’t, P.H.S.]. Mol Cell. 2014 11 20;56(4):595–607. PubMed PMID: 25458846; PubMed Central PMCID: PMC4255362. eng. 25458846 10.1016/j.molcel.2014.09.023 PMC4255362

[9] Kachhap SK, Faith D, Qian DZ, et al The N-Myc down regulated Gene1 (NDRG1) Is a Rab4a effector involved in vesicular recycling of E-cadherin [Research Support, N.I.H., Extramural Research Support, Non-U.S. Gov’t]. PloS One. 2007 9 5;2(9):e844 PubMed PMID: 17786215; PubMed Central PMCID: PMC1952073. eng.electronic. 17786215 10.1371/journal.pone.0000844 PMC1952073

[10] Jiang JH, Liu C, Cheng H, et al Epithelial-mesenchymal transition in pancreatic cancer: is it a clinically significant factor? [Research Support, Non-U.S. Gov’t Review]. Biochim Biophys Acta. 2015 1;1855(1):43–49. PubMed PMID: 25432020; eng. 25432020 10.1016/j.bbcan.2014.11.004

[11] Ueki T, Toyota M, Sohn T, et al Hypermethylation of multiple genes in pancreatic adenocarcinoma [Research Support, Non-U.S. Gov’t]. Cancer Res. 2000 4 1;60(7):1835–1839. PubMed PMID: 10766168; eng. 10766168

[12] Iwatani M, Ikegami K, Kremenska Y, et al Dimethyl sulfoxide has an impact on epigenetic profile in mouse embryoid body [Research Support, Non-U.S. Gov’t]. Stem Cells. 2006 11;24(11):2549–2556. PubMed PMID: 16840553; eng. 16840553 10.1634/stemcells.2005-0427

[13] Sonnichsen B, De Renzis S, Nielsen E, et al Distinct membrane domains on endosomes in the recycling pathway visualized by multicolor imaging of Rab4, Rab5, and Rab11 [Research Support, Non-U.S. Gov’t]. J Cell Biol. 2000 5 15;149(4):901–914. PubMed PMID: 10811830; PubMed Central PMCID: PMC2174575. eng. 10811830 10.1083/jcb.149.4.901 PMC2174575

[14] Zerial M, McBride H Rab proteins as membrane organizers [Review]. Nature Reviews Molecular Cell Biology. 2001 2;2(2):107–117. PubMed PMID: 11252952; eng. . 11252952 10.1038/35052055

[15] Agoulnik IU, Hodgson MC, Bowden WA, et al INPP4B: the new kid on the PI3K block [Research Support, N.I.H., Extramural Research Support, U.S. Gov’t, Non-P.H.S. Research Support, U.S. Gov’t, P.H.S. Review]. Oncotarget. 2011 4;2(4):321–328. PubMed PMID: 21487159; PubMed Central PMCID: PMC3248162. eng. 21487159 10.18632/oncotarget.260 PMC3248162

[16] Thiery JP Epithelial-mesenchymal transitions in tumour progression [Review]. Nature Reviews Cancer. 2002 6;2(6):442–454. PubMed PMID: 12189386; eng. . 12189386 10.1038/nrc822

[17] Ma K, Cheung SM, Marshall AJ, et al PI(3,4,5)P3 and PI(3,4)P2 levels correlate with PKB/akt phosphorylation at Thr308 and Ser473, respectively; PI(3,4)P2 levels determine PKB activity [Research Support, Non-U.S. Gov’t]. Cell Signal. 2008 4;20(4):684–694. PubMed PMID: 18249092; eng. 18249092 10.1016/j.cellsig.2007.12.004

[18] Klymkowsky MW, Savagner P Epithelial-mesenchymal transition: a cancer researcher’s conceptual friend and foe [Research Support, N.I.H., Extramural Research Support, Non-U.S. Gov’t Review]. Am J Pathol. 2009 5;174(5):1588–1593. PubMed PMID: 19342369; PubMed Central PMCID: PMC2671246. eng. 19342369 10.2353/ajpath.2009.080545 PMC2671246

[19] Liang S, Salas T, Gencaslan E, et al Tuberin-deficiency downregulates N-cadherin and upregulates vimentin in kidney tumor of TSC patients [Research Support, Non-U.S. Gov’t Research Support, U.S. Gov’t, Non-P.H.S.]. Oncotarget. 2014 8 30;5(16):6936–6946. PubMed PMID: 25149531; PubMed Central PMCID: PMC4196174. eng. 25149531 10.18632/oncotarget.2206 PMC4196174

[20] De Craene B, Berx G Regulatory networks defining EMT during cancer initiation and progression [Research Support, Non-U.S. Gov’t Review]. Nature Reviews Cancer. 2013 2;13(2):97–110. PubMed PMID: 23344542; eng. . 23344542 10.1038/nrc3447

[21] Frixen UH, Behrens J, Sachs M, et al E-cadherin-mediated cell-cell adhesion prevents invasiveness of human carcinoma cells [Research Support, Non-U.S. Gov’t]. J Cell Biol. 1991 4;113(1):173–185. PubMed PMID: 2007622; PubMed Central PMCID: PMC2288921. eng. 2007622 10.1083/jcb.113.1.173 PMC2288921

[22] Lieber M, Mazzetta J, Nelson-Rees W, et al Establishment of a continuous tumor-cell line (panc-1) from a human carcinoma of the exocrine pancreas. International Journal of Cancer. 1975 5 15;15(5):741–747. PubMed PMID: 1140870; eng. 1140870 10.1002/ijc.2910150505

[23] Kyriazis AP, McCombs WB 3rd, Sandberg AA, et al Establishment and characterization of human pancreatic adenocarcinoma cell line SW-1990 in tissue culture and the nude mouse [Research Support, U.S. Gov’t, P.H.S.]. Cancer Res. 1983 9;43(9):4393–4401. PubMed PMID: 6871872; eng. 6871872

[24] Tan MH, Nowak NJ, Loor R, et al Characterization of a new primary human pancreatic tumor line [Research Support, U.S. Gov’t, P.H.S.]. Cancer Invest. 1986;4(1):15–23. PubMed PMID: 3754176; eng. 3754176 10.3109/07357908609039823

[25] Chen WH, Horoszewicz JS, Leong SS, et al Human pancreatic adenocarcinoma: in vitro and in vivo morphology of a new tumor line established from ascites [Research Support, U.S. Gov’t, P.H.S.]. In Vitro. 1982 1;18(1):24–34. PubMed PMID: 7182348; eng. 7182348 10.1007/BF02796382

[26] Fujita Y, Krause G, Scheffner M, et al Hakai, a c-Cbl-like protein, ubiquitinates and induces endocytosis of the E-cadherin complex [Research Support, Non-U.S. Gov’t]. Nat Cell Biol. 2002 3;4(3):222–231. PubMed PMID: 11836526; eng. 11836526 10.1038/ncb758

[27] Le TL, Yap AS, Stow JL Recycling of E-cadherin: a potential mechanism for regulating cadherin dynamics [Research Support, Non-U.S. Gov’t]. J Cell Biol. 1999 7 12;146(1):219–232. PubMed PMID: 10402472; PubMed Central PMCID: PMC2199726. eng. 10402472 PMC2199726

[28] Vicinanza M, D’Angelo G, Di Campli A, et al Phosphoinositides as regulators of membrane trafficking in health and disease [Research Support, Non-U.S. Gov’t Review]. Cellular and Molecular Life Sciences: CMLS. 2008 9;65(18):2833–2841. PubMed PMID: 18726176; eng. 18726176 10.1007/s00018-008-8353-2 PMC11131623

[29] Gruenberg J The endocytic pathway: a mosaic of domains [Comparative Study Research Support, Non-U.S. Gov’t Review]. Nature Reviews Molecular Cell Biology. 2001 10;2(10):721–730. PubMed PMID: 11584299; eng. . 11584299 10.1038/35096054

[30] Ross ME, Zhou X, Song G, et al Classification of pediatric acute lymphoblastic leukemia by gene expression profiling [Research Support, Non-U.S. Gov’t Research Support, U.S. Gov’t, P.H.S.]. Blood. 2003 10 15;102(8):2951–2959. 10.1182/blood-2003-01-0338. PubMed PMID: 12730115; eng. 12730115 10.1182/blood-2003-01-0338

[31] Wang N, Stamenovic D Contribution of intermediate filaments to cell stiffness, stiffening, and growth [Research Support, U.S. Gov’t, P.H.S.]. American Journal Physiology Cell Physiology. 2000 7;279(1):C188–C194. PubMed PMID: 10898730; eng. 10.1152/ajpcell.2000.279.1.C188 10898730

[32] Prahlad V, Yoon M, Moir RD, et al Rapid movements of vimentin on microtubule tracks: kinesin-dependent assembly of intermediate filament networks [Research Support, Non-U.S. Gov’t Research Support, U.S. Gov’t, P.H.S.]. J Cell Biol. 1998 10 05;143(1):159–170. PubMed PMID: 9763428; PubMed Central PMCID: PMC2132817. eng. 9763428 10.1083/jcb.143.1.159 PMC2132817

[33] Hirokawa N, Noda Y, Tanaka Y, et al Kinesin superfamily motor proteins and intracellular transport [Research Support, Non-U.S. Gov’t Review]. Nature Reviews Molecular Cell Biology. 2009 10;10(10):682–696. PubMed PMID: 19773780; eng. 19773780 10.1038/nrm2774

[34] Li Chew C, Lunardi A, Gulluni F, et al In Vivo Role of INPP4B in Tumor and Metastasis Suppression through Regulation of PI3K-AKT Signaling at Endosomes [Research Support, N.I.H., Extramural Research Support, Non-U.S. Gov’t]. Cancer Discov. 2015 7;5(7):740–751. PubMed PMID: 25883022; PubMed Central PMCID: PMC4497843. eng. 25883022 10.1158/2159-8290.CD-14-1347 PMC4497843

[35] Brueckner B, Garcia BR, Siedlecki P, et al Epigenetic reactivation of tumor suppressor genes by a novel small-molecule inhibitor of human DNA methyltransferases [Research Support, Non-U.S. Gov’t]. Cancer Res. 2005 7 15;65(14):6305–6311. PubMed PMID: 16024632; eng. 16024632 10.1158/0008-5472.CAN-04-2957

[36] Stresemann C, Brueckner B, Musch T, et al Functional diversity of DNA methyltransferase inhibitors in human cancer cell lines [Research Support, Non-U.S. Gov’t]. Cancer Res. 2006 3 1;66(5):2794–2800. PubMed PMID: 16510601; eng. 16510601 10.1158/0008-5472.CAN-05-2821

[37] Smiraglia DJ, Rush LJ, Fruhwald MC, et al Excessive CpG island hypermethylation in cancer cell lines versus primary human malignancies [Comparative Study Research Support, Non-U.S. Gov’t Research Support, U.S. Gov’t, P.H.S.]. Hum Mol Genet. 2001 6 15;10(13):1413–1419. PubMed PMID: 11440994; eng. 11440994 10.1093/hmg/10.13.1413

[38] Chew CL, Chen M, Pandolfi PP Endosome and INPP4B [Editorial]. Oncotarget. 2016 1 5;7(1):5–6. PubMed PMID: 26700619; PubMed Central PMCID: PMC4807978. eng. . 26700619 10.18632/oncotarget.6663 PMC4807978

[39] Werner S, Stenzl A, Pantel K, et al Expression of Epithelial Mesenchymal Transition and Cancer Stem Cell Markers in Circulating Tumor Cells. Adv Exp Med Biol. 2017;994:205–228. PubMed PMID: 28560676; eng. . 28560676 10.1007/978-3-319-55947-6_11

[40] Tondeur S, Pangault C, Le Carrour T, et al Expression map of the human exome in CD34+ cells and blood cells: increased alternative splicing in cell motility and immune response genes [Research Support, Non-U.S. Gov’t]. PloS One. 2010 2 1;5(2):e8990 PubMed PMID: 20126548; PubMed Central PMCID: PMC2813875. eng.electronic. 20126548 10.1371/journal.pone.0008990 PMC2813875

