# Supplementary figures and images for: Inositol polyphosphate-4-phosphatase type II plays critical roles in the modulation of cadherin-mediated adhesion dynamics of pancreatic ductal adenocarcinomas
Source: Cell Adh Migr. 2018 Aug 19;12(6):548–63. doi: 10.1080/19336918.2018.1491496 (PMC6363046; doi:10.1080/19336918.2018.1491496)

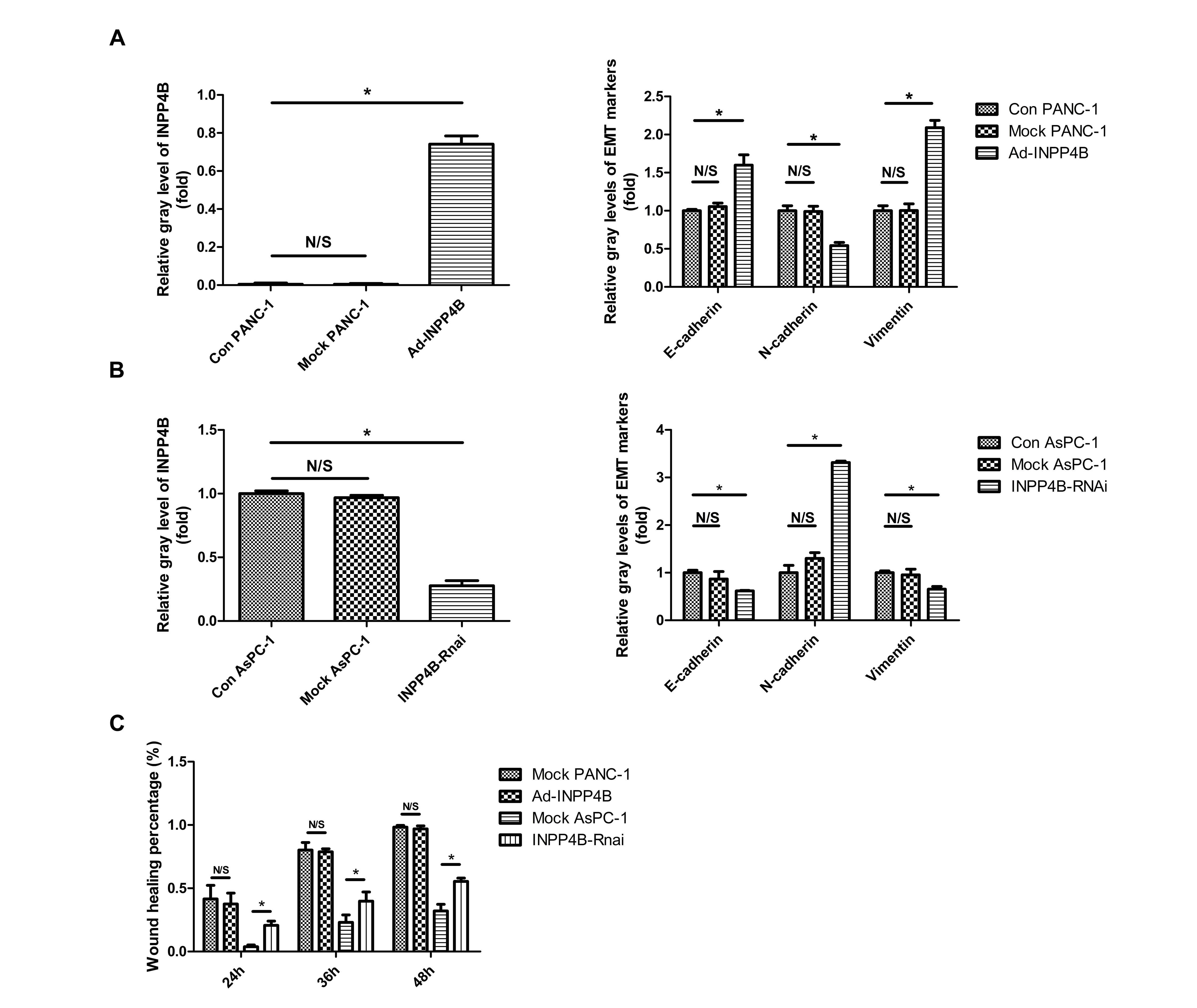

Supplement: Supplemental Material [file kcam-12-06-1491496-s001.zip › Supp/2017CAM0068R-f08-z-bw.tif]

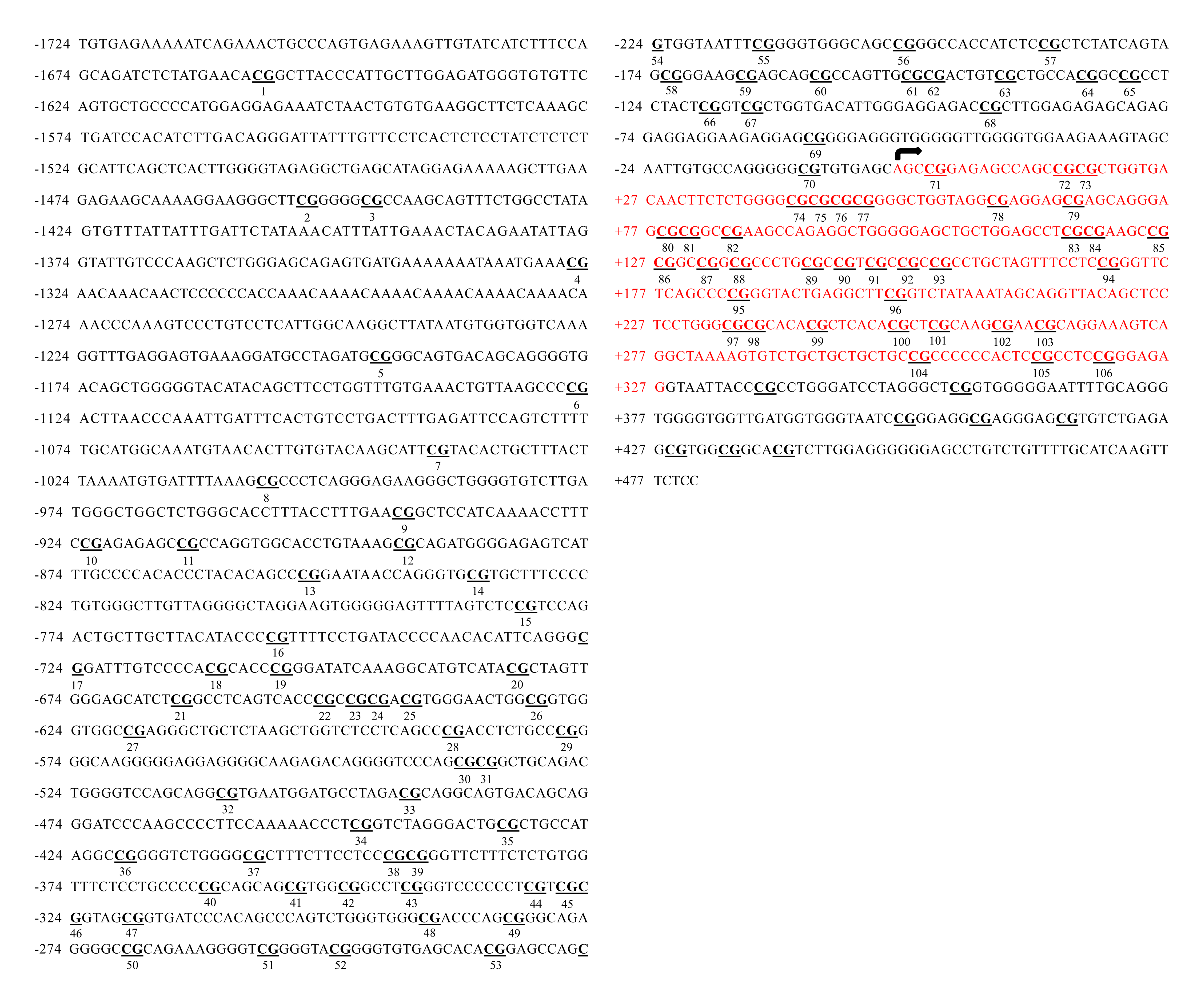

Supplement: Supplemental Material [file kcam-12-06-1491496-s001.zip › Supp/2017CAM0068R-f09-z-4c.tif]
