# Supplementary material for: Inositol polyphosphate-4-phosphatase type II plays critical roles in the modulation of cadherin-mediated adhesion dynamics of pancreatic ductal adenocarcinomas
Source: Cell Adh Migr. 2018 Aug 19;12(6):548–63. doi: 10.1080/19336918.2018.1491496 (PMC6363046; doi:10.1080/19336918.2018.1491496)
Supplement: Supplemental Material [file kcam-12-06-1491496-s001.zip › Supp/2017CAM0068R-s01.docx]

**Dear editor：**

Thank you very much for all the efficient works you have done for our manuscript (No. 2017CAM0068). We feel extremely sorry and apologize for our inexperience and low efficiency in conducting supplementary experiments and revising the manuscript. We wish it were not too late for us to send back the new manuscript. The up-to-date contact information for all authors, including titles, credentials, phone numbers and e-mail addresses were listed in a table as followed. I have informed all coauthors of the status of this article.

| **Author name** | **titles** | **credentials** | **phone numbers** | **e-mail addresses** |
| --- | --- | --- | --- | --- |
| Bin Zhang | Dr. | Ph.D. | +8615701571946 | binzhang_0021@outlook.com |
| Rong Liu | Dr. | Ph.D. | +8613801150988 | liurong301@126.com |
| Weidong Wang | Dr. | Ph.D. | +8613331029859 | 462707177@qq.com |
| Chonghui Li | Dr. | Ph.D. | +8613522351926 | betterLCH@163.com |

We have finished the revision according to the comments and requests of editor and peer reviewers. The following materials are the major explaining for all the modification, updating and supplement we have done under the guidance of reviewers.

Sincerely yours,

Rong Liu, Ph.D.

2nd Department of Hepatobiliary Surgery, Chinese PLA General Hospital

Beijing, China

**To dear Professor Reviewer #1:**

We are grateful for the all the remarkable comments you proposed to our study, and we conducted some supplementary works under your tutorship.

The following materials are the major explaining for all the modification, updating and supplement we have done.

1. Thank you very much for reminding us of the partial EMT. We used ‘partial EMT’ as a search term you suggested to us, and went through the resulting review articles. Reference [1, 2] depicts that the poorly differentiated state of many carcinoma cells may reflect a dedifferentiation rather than a transdifferentiation process. From this perspective, transformed epithelial progenitors may simply fail to differentiate normally, rather than differentiate into mesenchymal cells. We realize that the loss of cell-cell adhesion does not make an EMT, or just a partial EMT.
2. Accordingly, additional approaches were utilized to verify the potential links between INPP4B and EMT as a pathophysiological process, including light microscopy, wound healing assay and RT-qPCR analysis of EMT-associated transcription factors. The morphologic characteristic of PDAC cells observed under phase contrast microscope was not significantly changed when INPP4B expression altered. Specially, mock control PANC-1 cells displayed a mesenchymal-like morphology, and Ad-INPP4B infected PANC-1 cells were still elongated in shape (Fig. 3C). Mock control AsPC-1 cells were observed to form tight colonies. After INPP4B knockdown, cells were more scattered but without evident shape change (Fig. 3D). The scanty cytoplasm and large nucleus of PANC-1 and AsPC-1 made it hard to discern whether the cell shape seen was unchanged or not. In terms of wound closing, INPP4B overexpression did not change the migratory capacity of PANC-1 cells, whereas the interference of INPP4B in AsPC-1 led to an enhancement in wound healing ability (Fig 4B; Fig. SC). RT-qPCR analysis indicated that the expression of ZEB1 and SNAIL2 were notably downregulated in Ad-INPP4B group, whereas INPP4B interference in AsPC-1 resulted in a significant increase of ZEB1, SNAIL1 and TWIST1 (Fig. 3A, B). These findings confirmed a link between the expression of INPP4B and partial reversion of mesenchymal status or just the cadherin switch in PANC-1 and AsPC-1. The model we proposed was not a perfect EMT/MET, but at least a transient regulation of cell-cell adhesion structures. Perhaps inhibition of cell proliferation or proteases that degraded extracellular matrix was involved in INPP4B-oriented suppression of PANC-1 invasion but not migration. All the above words were depicted in the revised manuscript. Therefore, we have changed the manuscript title to ‘Inositol polyphosphate-4-phosphatase type II plays critical roles in the modulation of cadherin-mediated adhesion dynamics of pancreatic ductal adenocarcinomas’ and avoided any affirmative statement that INPP4B regulates EMT in PDAC cells. EMT was removed from the storyline of this study.

In addition, two pairs of primers were designed for ZEB2 and none of them can amplify ZEB2 fragment from PANC-1 and AsPC-1. The cDNA from TPC-1 and A498 cell lines was served as positive control. It denoted the expression of ZEB2 in PANC-1 and AsPC-1 was too scarce to be detected. Therefore, we did not further analyze it.

(1: DNA ladder; 2: PANC-1, primer 1; 3: AsPC-1, primer 1; 4: TPC-1, primer 1; 5: A498, primer 1; 6: blank control; 7: PANC-1, primer 2; 8: AsPC-1, primer 2; 9: TPC-1, primer 2; 10: A498, primer 2).

3. Upon your suggestion, we selected the AKT activator (SC79) and inhibitor (MK-2206) for further analysis. By western blot, we found that the INPP4B overexpression in PANC-1 contributed more to Ser473 dephosphorylation than to Thr308, and INPP4B knockdown in AsPC-1 only resulted in Ser473 phosphorylation and activation of AKT (Fig. 2A). Therefore, the Ad-INPP4B cells were subjected to SC79 treatment whereas the INPP4B-Rnai cells were subjected to MK-2206 incubation. SC79 treatment led to a significant increase in p-AKT at Ser473 and Thr308 compared to DMSO-treated cells, which contributed to the impairment of INPP4B function on EMT marker turnover. MK-2206 treatment in INPP4B-siRNA transfected AsPC-1 simulated the effect of INPP4B on p-AKT (Ser473) and compensated for the adverse impact of INPP4B loss on cadherin and Vim expression compared to DMSO-treated cells (Fig. 2B). We concluded that INPP4B was an tumor suppressor in PDAC which attenuates aberrant AKT activation, and the EMT marker expression was regulated by INPP4B in an AKT-dependent manner. All the above words were depicted in the revised manuscript.
4. In contrast to most studies, Ncad and Vim show discorant behaviour in response to manipulation of INPP4B. We interpreted as that ‘the limited variation of Vim in our study could not reach that far as overexpression do, which we thought would be a physiological response to INPP4B alternation rather than pathologic effect and required further investigation. Functions of Vim contributed to the construction of cytoskeleton architecture within cells by interacting with microtubules [3]. As showed by boxed areas of Fig.1G, Ecad staining transformed into a filamentous appearance when INPP4B was overexpressed. We suggest that Vim should be motivated and recruited as a candidate for cross-bridging microtubules and Ecad containing vesicles if INPP4B was upregulated’. The effect of p-AKT on Vim expression was in consistence with a similar unusual study in leiomyosarcoma [4]. All the above words were depicted in the revised manuscript.
5. **Reference:**

1.	Klymkowsky MW, Savagner P. Epithelial-mesenchymal transition: a cancer researcher's conceptual friend and foe [Research Support, N.I.H., Extramural Research Support, Non-U.S. Gov't Review]. The American journal of pathology. 2009 May;174(5):1588-93. doi: 10.2353/ajpath.2009.080545. PubMed PMID: 19342369; PubMed Central PMCID: PMC2671246. eng.

2.	De Craene B, Berx G. Regulatory networks defining EMT during cancer initiation and progression [Research Support, Non-U.S. Gov't Review]. Nature reviews Cancer. 2013 Feb;13(2):97-110. doi: 10.1038/nrc3447. PubMed PMID: 23344542; eng.

3.	Wang N, Stamenovic D. Contribution of intermediate filaments to cell stiffness, stiffening, and growth [Research Support, U.S. Gov't, P.H.S.]. American journal of physiology Cell physiology. 2000 Jul;279(1):C188-94. PubMed PMID: 10898730; eng.

4.	Liang S, Salas T, Gencaslan E, et al. Tuberin-deficiency downregulates N-cadherin and upregulates vimentin in kidney tumor of TSC patients [Research Support, Non-U.S. Gov't Research Support, U.S. Gov't, Non-P.H.S.]. Oncotarget. 2014 Aug 30;5(16):6936-46. doi: 10.18632/oncotarget.2206. PubMed PMID: 25149531; PubMed Central PMCID: PMC4196174. eng.

We get into some dilemma there in terms of financial support for this study, and we could only complement the study for that much. Please allow me to extend heartfelt thanks again for your help on our works.

Sincerely yours,

Rong Liu, Ph.D.

2nd Department of Hepatobiliary Surgery, Chinese PLA General Hospital

Beijing, China

**To dear Professor Reviewer #2:**

We are grateful for the all the remarkable comments you proposed to our study, and we conducted some complementary works under your tutorship.

The following materials are the major explaining for all the modification, updating and supplement we have done.

1. **Comment:** Figure 1: Figure legend needs elaboration. Important details are missing in the figure legend and the corresponding section in the methodology. For instance, the number of biological and technical replicates is missing. Is it sufficient to draw a biological conclusion ?

**Revision:** We hides the results of pre-experiment including screening of siRNA with best interference efficiency and the epi-fluorescence imaging of EGFP expression from transfected vectors, and we have done some compensatory works to enrich our study under your tutorship. We combined the previous Fig. 1 and Fig. 2 and elaborated new Figure 1 legend as ‘**Figure 1. INPP4B expression in four PDAC cell lines and its impact on EMT marker in PANC-1 and AsPC-1.** Relative INPP4B expression level in four pancreatic cancer cell lines was quantified by western blot (A) and RT-qPCR (B) and listed as follows : AsPC-1 > BxPC-3 > SW1990 > PANC-1 (All *P* < 0.05). The PANC-1 with relatively minimal INPP4B level was chosen for overexpression assay, and AsPC-1 with the highest INPP4B level among was selected for knockdown use. INPP4B overexpression induced upregulation of Ecad and Vim with downregulated Ncad in PANC-1 compared to that of the control in protein (C) and transcript levels (D). Knockdown of INPP4B in AsPC-1 led to reduction of Ecad and Vim with increased Ncad compared to control group (E, F). These results were further substantiated by immunofluorescence analysis for Ecad (G, top panel), Ncad (middle panel) and Vim (bottom panel) expression. Scale bar in images = 20 μm. The quantitative results of western blot and PCR were analyzed basing on gray-level ratio of target band and 2^-ΔΔCt^ method. GAPDH was used as housekeeping gene for all data normalization. Results show mean ± SD (n = 3). N/S and * indicate *P* > 0.05 and *P* < 0.05. The boxes and arrowheads in merge images indicate regions of interest.’

2. **Comment:** Figure 1A, B do not justify the clustering of the cell lines into 2 groups especially the difference between BxPC3 and SW1990; Figure 1B and methods subsection ‘quantification of INPP4B in PDAC cell lines’ are missing the quantification method used in the qRT-PCR assay. The house keeping gene used in the relative quantification of gene expression is missing. The relative quantification to AsPC-1 needs explicit disclosure in the legend. The quantitative interpretation and statistical significance of the disparity in the gene expression is lacking. For example, is the difference in INPP4B expression between AsPC-1 and BxPC3 significant? How much is the difference between BxPC3 and SW1990? Is it significant ?

**Revision:** We rescheduled the materials and methods subsection. RT-qPCR and western blot were introduced independently. In RT-qPCR, the comparative cycle threshold (2^-ΔΔCt^) method mapped the relative expression of target gene. GAPDH was used as housekeeping gene for all data normalization. In western blot, the gray-level of target band relative to GAPDH in each group was established by Quantity One software. We compared the INPP4B expression in these cell lines respectively, and the level was listed: AsPC-1 > BxPC-3 > SW1990 > PANC-1 (All P < 0.05). Results show mean ± SD (n = 3). All the above words were depicted in the revised manuscript.

3. **Comment:** Figure 1C: the authors confused the dark field imaging with epi-fluorescence imaging of EGFP expression from transfected vectors. Contrast with mock and control is needed as in Fig 1D. Authors need to quantify the expression of INPP4B after the successful transfection.

**Revision:** We feel sorry for making this mistake, and we hided these pre-experiment results which were not necessary. The expression of INPP4B after the successful transfection was revealed in new Figure 1 in Ad-INPP4B group.

4. **Comment:** Figure 2: The EMT turnover statement is not justified by the expression of vimentin. The change in cadherin proteins is not sufficient. Investigation of cell morphology using light microscopy is needed. Imaging of epithelial and mesenchymal markers expression using confocal fluorescence imaging is needed. Comprehensive expression analysis of a number of transcription factors involved in the induction of EMT or MET upon regulation of INPP4B is needed. Suggested transcription factors include SNAL1, SNAL2, ZEB1, ZEB2 and TWIST. Number of replicates used in this experiment is not disclosed.

**Revision:** Thank you very much for reminding us that the loss of cell-cell adhesion does not sufficiently make an EMT, or just a partial EMT. Accordingly, additional approaches were utilized to verify the potential links between INPP4B and EMT as a pathophysiological process, including light microscopy, wound healing assay and RT-qPCR analysis of EMT-associated transcription factors. The morphologic characteristic of PDAC cells observed under phase contrast microscope was not significantly changed when INPP4B expression altered. Specially, mock control PANC-1 cells displayed a mesenchymal-like morphology, and Ad-INPP4B infected PANC-1 cells were still elongated in shape (Fig. 3C). Mock control AsPC-1 cells were observed to form tight colonies. After INPP4B knockdown, cells were more scattered but without evident shape change (Fig. 3D). The scanty cytoplasm and large nucleus of PANC-1 and AsPC-1 made it hard to discern whether the cell shape seen was unchanged or not. In terms of wound closing, INPP4B overexpression did not change the migratory capacity of PANC-1 cells, whereas the interference of INPP4B in AsPC-1 led to an enhancement in wound healing ability (Fig 4B; Fig. SC). RT-qPCR analysis indicated that the expression of ZEB1 and SNAIL2 were notably downregulated in Ad-INPP4B group, whereas INPP4B interference in AsPC-1 resulted in a significant increase of ZEB1, SNAIL1 and TWIST1 (Fig. 3A, B). These findings confirmed a link between the expression of INPP4B and partial reversion of mesenchymal status or just the cadherin switch in PANC-1 and AsPC-1. The model we proposed was not a perfect EMT/MET, but at least a transient regulation of cell-cell adhesion structures. Perhaps inhibition of cell proliferation or proteases that degraded extracellular matrix was involved in INPP4B-oriented suppression of PANC-1 invasion but not migration. All the above words were depicted in the revised manuscript. Therefore, we have changed the manuscript title to ‘Inositol polyphosphate-4-phosphatase type II plays critical roles in the modulation of cadherin-mediated adhesion dynamics of pancreatic ductal adenocarcinomas’ and avoided any affirmative statement that INPP4B regulates EMT in PDAC cells. EMT was removed from the storyline of this study.

In addition, two pairs of primers were designed for ZEB2 and none of them can amplify ZEB2 fragment from PANC-1 and AsPC-1. The cDNA from TPC-1 and A498 cell lines was served as positive control. It denoted the expression of ZEB2 in PANC-1 and AsPC-1 was too scarce to be detected. Therefore, we did not further analyze it.

(1: DNA ladder; 2: PANC-1, primer 1; 3: AsPC-1, primer 1; 4: TPC-1, primer 1; 5: A498, primer 1; 6: blank control; 7: PANC-1, primer 2; 8: AsPC-1, primer 2; 9: TPC-1, primer 2; 10: A498, primer 2).

5. **Comment:** Text ‘Moreover, PANC-1 was more prone to metastasis than AsPC-1 in vitro’: statement lacks reference to figure or literature. The term ‘metastasis’ has a multi-phase implication in ‘*in vivo*’ settings which is not tested herein. I suggest sticking to the term ‘*in vitro* invasion’.

**Revision:** We read too much into the result of *in vitro* invasion assay and deleted the text ‘Moreover, PANC-1 was more prone to metastasis than AsPC-1 in vitro’. I did stick to the term ‘*in vitro* invasion’ in the new manuscript.

6. **Comment:** Figure 4A: The conclusion drawn about E-cadherin expression is not justified by the results. There is no significant change in the susceptibility of E-cadherin to CHX between mock and test conditions. The text claim that there is a difference between the mock and the transfected cells. The claim is clearly false.

**Revision:** We went through the references [1, 2] and figured out that Ecad has a half life of 5 h~10 h. We re-conducted this assay and prolonged the CHX treatment duration to 7 h. After accurately normalized to GAPDH, we concluded that PANC-1 overexpressing INPP4B showed a less decrease in Ecad protein levels as compared to mock infected and uninfected cells (*P* < 0.05; Fig. 5A). This illustrated Ecad could be stabilized by INPP4B. All the above words were depicted in the revised manuscript.

7. **Comment:** Figure 5 legend is severely insufficient. Authors need to give a brief description of the procedure, how the quantification was performed and what the co-localization indicates.

**Revision:** After discontinuous sucrose gradient centrifugation and protein extraction with methanol/chloroform, western blot of equal amount of proteins from resulting cellular fractions showed that the INPP4B and Ecad were mainly precipitated from Rab5 and 11 positive parts (Fig. 6B). When normalized by Rab5 and 11 in EE and RE fractions, the early and sorting pools of Ecad were enriched by INPP4B overexpression comparing to that of the control. We also normalized the corresponding Ecad to Rab7 in LE fraction and observed that the degradation pools of Ecad in Ad-INPP4B cells decreased. Moreover, the Rab11/Rab5 ratio in EE and RE fractions was elevated in Ad-INPP4B cells which could be interpreted as INPP4B overexpression promoted transport and recycling of membrane protein (All P < 0.05; Fig. 6C). We concluded that INPP4B increases Ecad at early and recycling endosomes and suppresses its late endosomal lodging. However, we did not further purify RE from target fractions via immunoisolation using Rab11 antibody bound to immunomagnetic, which confined the level of evidence. The new figure legend was elaborated in manuscript ‘**Figure 6. INPP4B co-localizes and co-fractionates with endocytic Ecad.** (A) The dynamics of Ecad recycling relating to INPP4B in RG108-treated PANC-1 cells were investigated via immunofluorescence analysis at different time points after calcium chelation. Immunostaining of Ecad (red) and INPP4B (green) were shown in separate channels. The boxes, arrows and arrowheads in merge images indicate regions of interest. (B) PANC-1 cells were subjected to a discontinuous sucrose density gradient centrifugation, and the proteins from resulting cell fractions were loaded for western blot. (C) The Ecad and INPP4B blots were normalized to Rab5 and 11 in EE and RE, as well as Rab7 in LE, to reveal the endosomal contents respectively. The Rab11/Rab5 ratio in EE and RE was calculated to reflect the relative recycling activity relating to INPP4B overexpression. PNS: postnuclear supernatant; EE: early endosomes; RE: recycling endosomes; LE: late endosomes; HM: heavy membranes. The results of western blot were analyzed basing on gray-level ratio of target band. Results show mean ± SD (n = 3). * indicate *P* < 0.05.’ All the above words were depicted in the revised manuscript.

8. **Comment:** RG108 experiment needs confirmation of inhibition of DNA methylation globally or, preferably, in the proximity of the INPP4B gene at least.

**Revision:** In order to avoid false-positive result from INPP4B overexpression in double-labeling immunofluorescence of Ecad and INPP4B, we sought for some reagents that might upregulate INPP4B and Ecad simultaneously in PANC-1, and the reference [3] offered us some illuminations. The preliminary experiment showed that RG108 could increase both the proteins in PANC-1. Accordingly, the PANC-1 cells were pretreated with RG108 rather than Ad-INPP4B to discern the possible co-localization. RG108 treatment enabled the immunostaining of both the proteins in PANC-1 to monitor the recycling dynamic of Ecad relating to INPP4B. Under your tutorship, MassARRAY quantitative methylation analysis of INPP4B gene promoter region in PANC-1 was conducted (Fig. S2), and we found that the methylation scale of almost 91% of the total CpG units evaluated was below 20%. The highest methylation level (100%) was only found at the 7th CpG sites. When treated with RG108, though the methylation levels of certain CpG units declined (All *P* < 0.05; Fig. 5E), the average methylation percentage across total CpG sites evaluated was not significantly reduced (*P* > 0.05; Fig. 5F). Therefore, RG108 upregulated INPP4B expression without apparently inducing DNA demethylation of its promoter region. It might be ascribed to the intrinsic hypomethylation of overall INPP4B promoter region in PANC-1. Promoter region hypermethylation is not a mechanism for the silencing of INPP4B in PANC-1. All the above words were depicted in the revised manuscript.

9. **Comment:** The claim that INPP4B-mediated regulation of Ecad is epigenetic needs further investigation. Histone modification and transcription factor involvement upon EMT induction in PDAC cell model(s) is needed.

**Revision:** We selected the AKT activator (SC79) and inhibitor (MK-2206) for further analysis. By western blot, we found that the INPP4B overexpression in PANC-1 contributed more to Ser473 dephosphorylation than to Thr308, and INPP4B knockdown in AsPC-1 only resulted in Ser473 phosphorylation and activation of AKT (Fig. 2A). Therefore, the Ad-INPP4B cells were subjected to SC79 treatment whereas the INPP4B-Rnai cells were subjected to MK-2206 incubation. SC79 treatment led to a significant increase in p-AKT at Ser473 and Thr308 compared to DMSO-treated cells, which contributed to the impairment of INPP4B function on Ecad turnover. MK-2206 treatment in INPP4B-siRNA transfected AsPC-1 simulated the effect of INPP4B on p-AKT (Ser473) and compensated for the adverse impact of INPP4B loss on Ecad expression compared to DMSO-treated cells (Fig. 2B). We concluded that INPP4B was an tumor suppressor in PDAC which attenuates aberrant AKT activation, and the Ecad expression was regulated by INPP4B in an AKT-dependent manner. We eliminated the claim that INPP4B-mediated regulation of Ecad was epigenetic, and we can conclude INPP4B was an upstream regulator of both Ecad in these pancreatic cancer cells. All the above words were depicted in the revised manuscript.

10. **Comment:** Figure 6: the linked figure is not a confocal image. May be it is confused with the linked figure 5 and figure 5 is missing. Although the article should have 8 figures. I could see up to figure 7G only. Apparently, figure 5 is missing and did all the mess.

**Revision:** We feel extremely sorry for having trouble in uploading detailed images and making these mistakes. We have confirmed the integrity of all figures in the new submission.

11. **Reference**

1.	Fujita Y, Krause G, Scheffner M, et al. Hakai, a c-Cbl-like protein, ubiquitinates and induces endocytosis of the E-cadherin complex [Research Support, Non-U.S. Gov't]. Nature	cell biology. 2002 Mar;4(3):222-31. doi: 10.1038/ncb758. PubMed PMID: 11836526; eng.

2.	Kachhap SK, Faith D, Qian DZ, et al. The N-Myc down regulated Gene1 (NDRG1) Is a Rab4a effector involved in vesicular recycling of E-cadherin [Research Support, N.I.H.,	Extramural Research Support, Non-U.S. Gov't]. PloS one. 2007 Sep 5;2(9):e844. doi: 10.1371/journal.pone.0000844. PubMed PMID: 17786215; PubMed Central PMCID:	PMC1952073. eng.

3.	Li Chew C, Lunardi A, Gulluni F, et al. In Vivo Role of INPP4B in Tumor and Metastasis Suppression through Regulation of PI3K-AKT Signaling at Endosomes [Research Support, N.I.H., Extramural Research Support, Non-U.S. Gov't]. Cancer discovery. 2015 Jul;5(7):740-51. doi: 10.1158/2159-8290.CD-14-1347. PubMed PMID: 25883022; PubMed Central PMCID: PMC4497843. eng.

We get into some dilemma there in terms of financial support for this study, and we could only complement the study for that much. Please allow me to extend heartfelt thanks again for your help on our works.

Sincerely yours,

Rong Liu, Ph.D.

2nd Department of Hepatobiliary Surgery, Chinese PLA General Hospital

Beijing, China
